# From water striders to water bugs: the molecular diversity of aquatic Heteroptera (Gerromorpha, Nepomorpha) of Germany based on DNA barcodes

**DOI:** 10.7717/peerj.4577

**Published:** 2018-05-02

**Authors:** Nadine Havemann, Martin M. Gossner, Lars Hendrich, Jèrôme Morinière, Rolf Niedringhaus, Peter Schäfer, Michael J. Raupach

**Affiliations:** 1 Fakultät V, Institut für Biologie und Umweltwissenschaften (IBU), Carl von Ossietzky Universität Oldenburg, Oldenburg, Lower Saxony, Germany; 2 German Centre of Marine Biodiversity, Senckenberg Nature Research Society, Wilhelmshaven, Lower Saxony, Germany; 3 Forest Entomology, Swiss Federal Institute for Forest, Snow and Landscape Research, Birmensdorf, Switzerland; 4 Sektion Insecta varia, SNSB-Bavarian State Collection of Zoology, Munich, Bavaria, Germany; 5 Taxonomic coordinator—German Barcode of Life (GBOL), SNSB-Bavarian State Collection of Zoology, Munich, Bavaria, Germany; 6 Department of Biology, Earth and Environmental Sciences, Carl von Ossietzky Universität Oldenburg, Oldenburg, Lower Saxony, Germany; 7 B.U.G.S. (Biologische Umwelt-Gutachten Schäfer), Telgte, North-Rhine Westphalia, Germany

**Keywords:** Aquatic insects, *Cymatia*, Mitochondrial DNA, Corixidae, *Plea*, Taxonomy, Freshwater, *Sigara*, GBOL, Cytochrome *c* oxidase subunit I

## Abstract

With about 5,000 species worldwide, the Heteroptera or true bugs are the most diverse taxon among the hemimetabolous insects in aquatic and semi-aquatic ecosystems. Species may be found in almost every freshwater environment and have very specific habitat requirements, making them excellent bioindicator organisms for water quality. However, a correct determination by morphology is challenging in many species groups due to high morphological variability and polymorphisms within, but low variability between species. Furthermore, it is very difficult or even impossible to identify the immature life stages or females of some species, e.g., of the corixid genus *Sigara*. In this study we tested the effectiveness of a DNA barcode library to discriminate species of the Gerromorpha and Nepomorpha of Germany. We analyzed about 700 specimens of 67 species, with 63 species sampled in Germany, covering more than 90% of all recorded species. Our library included various morphological similar taxa, e.g., species within the genera *Sigara* and *Notonecta* as well as water striders of the genus *Gerris*. Fifty-five species (82%) were unambiguously assigned to a single Barcode Index Number (BIN) by their barcode sequences, whereas BIN sharing was observed for 10 species. Furthermore, we found monophyletic lineages for 52 analyzed species. Our data revealed interspecific K2P distances with below 2.2% for 18 species. Intraspecific distances above 2.2% were shown for 11 species. We found evidence for hybridization between various corixid species (*Sigara*, *Callicorixa*), but our molecular data also revealed exceptionally high intraspecific distances as a consequence of distinct mitochondrial lineages for *Cymatia coleoptrata* and the pygmy backswimmer *Plea minutissima*. Our study clearly demonstrates the usefulness of DNA barcodes for the identification of the aquatic Heteroptera of Germany and adjacent regions. In this context, our data set represents an essential baseline for a reference library for bioassessment studies of freshwater habitats using modern high-throughput technologies in the near future. The existing data also opens new questions regarding the causes of observed low inter- and high intraspecific genetic variation and furthermore highlight the necessity of taxonomic revisions for various taxa, combining both molecular and morphological data.

## Introduction

Aquatic insects are the dominant invertebrate fauna element in most freshwater ecosystems and are enormously variable in morphology, development, physiology, and ecology (Lancaster & Downes, 2013; Dijkstra, Monaghan & Pauls, 2014). Among the hemimetabolous insects, the Heteroptera or true bugs comprise a significant and diverse component of the world’s aquatic insect biota (Polhemus & Polhemus, 2008). They are unique as a group because they comprise both aquatic and terrestrial species, whereas other taxa include only species that are aquatic during some life stage, e.g., mayflies, stoneflies, or dragonflies (Wesenberg-Lund, 1943; Lancaster & Downes, 2013; Gullan & Cranston, 2014). Two infraorders, the Gerromorpha and Nepomorpha, are considered as primarily aquatic (Polhemus & Polhemus, 2008; Lancaster & Downes, 2013; Gullan & Cranston, 2014; Henry, 2017). With more than 4,400 described species worldwide (Henry, 2017), aquatic Heteroptera are well-known for utilizing an exceptionally broad range of habitats, ranging from the marine and intertidal to the arctic and high alpine (Polhemus & Polhemus, 2008). They may be found in almost every freshwater biotope. Approximately 120 species of the Gerromorpha and 230 species of the Nepomorpha are known from the Palearctic region (Polhemus & Polhemus, 2008). For Germany, 47 species of the Nepomorpha and 22 species belonging to the Gerromorpha have been recorded so far (Wachmann, Melber & Deckert, 2006; Strauss & Niedringhaus, 2014).

**Figure 1:**
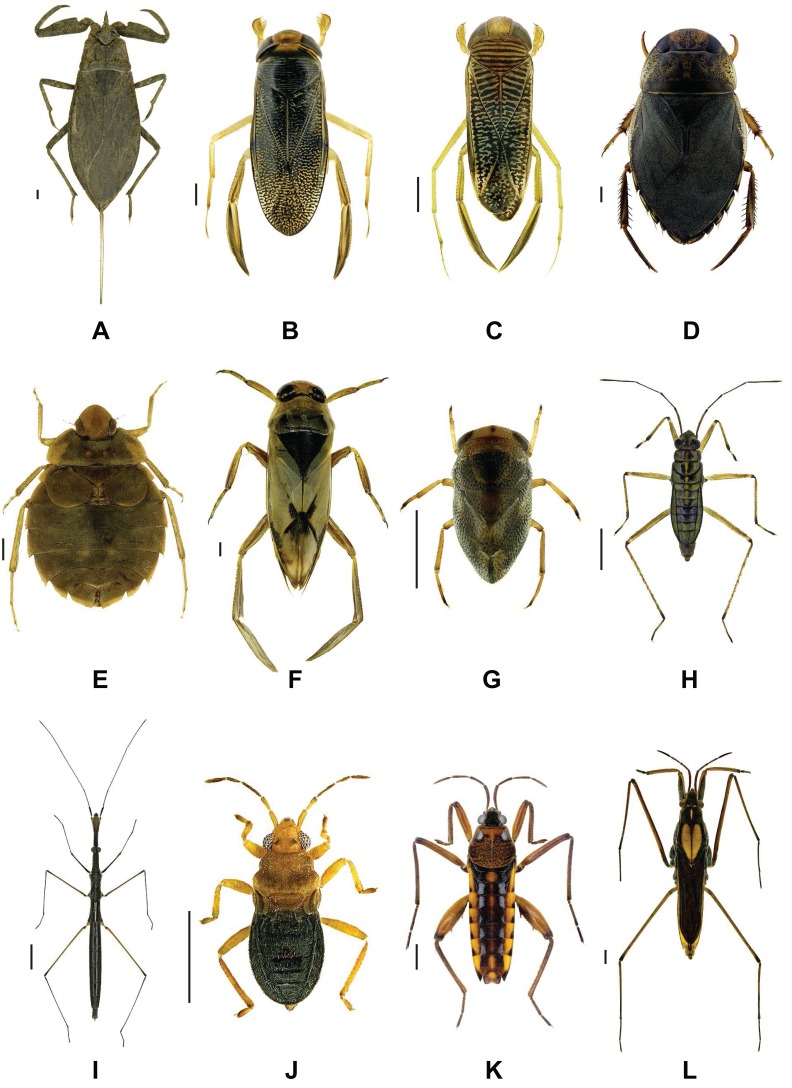
Representative images of analyzed aquatic bug species. (A) *Nepa cinerea* Linnaeus, 1758 (Nepidae), (B) *Corixa affinis* Leach, 1817 (Corixidae), (C) *Sigara* (*Subsigara*) *scotti* (Douglas & Scott, 1868) (Corixidae), (D) *Ilyocoris cimicoides* (Linnaeus, 1758) (Naucoridae), (E) *Aphelocheirus aestivalis* (Fabricius, 1794) (Aphelocheiridae), (F) *Notonecta viridis* Delcourt, 1909 (Notonectidae), (G) *Plea minutissima* Leach, 1817 (Pleidae), (H) *Mesovelia furcata* Mulsant & Rey, 1852 (Mesovelidae), (I) *Hydrometra gracilenta* Horváth, 1899 (Hydrometridae), (J) *Hebrus ruficeps* Thomson, 1871 (Hebridae), (K) *Velia caprai* Tamanini, 1947 (Velidae), (L) *Gerris costae* (Herrich-Schaeffer, 1840–1853) (Gerridae). Scale bars = 1 mm. All images were obtained from http://www.corisa.de.

Species of the Nepo- and Gerromorpha exhibit numerous morphological and ecological adaptations to their aquatic environment. For instance, nepomorphan true bugs have a streamlined body, natatorial legs and short antennas, whereas gerromorphan species are well-known for their long slender legs which operate as motive (middle leg) and rudder (hind legs), allowing them to operate on the water surface (Wesenberg-Lund, 1943; Andersen, 1982; Lancaster & Downes, 2013; Gullan & Cranston, 2014) (Fig. 1). Furthermore, a reduction, loss, and/or polymorphism of wings can be observed in many taxa, which is controlled by environmental conditions and genetic factors (Zera, Innes & Saks, 1983; Muraji, Miura & Nakasuji, 1989; Spence & Andersen, 1994). With the exception of the omnivorous Corixidae, all aquatic true bugs are predators, feeding on any organism that can be subdued by the injection of a venom cocktail consisting of various toxins and proteolytic enzymes (Polhemus & Polhemus, 2008). On the other hand they serve as important prey for numerous fish and other organisms at higher trophic levels (McCafferty, 1981; Peckarsky, 1982; Zimmermann & Spence, 1989; Hutchinson, 1993; Klecka, 2014; Boda et al., 2015).

Due to their general high abundance in many freshwater systems, their great value as bioindicators of water quality and their unique morphological and ecological specializations for exploiting specialized microhabitats, these groups has been in the focus of entomological and ecological research for a long time (Hufnagel, Bakonyi & Vásárhelyi, 1999; Polhemus & Polhemus, 2008; Whiteman & Sites, 2008; Skern, Zweimüller & Schiemer, 2010). Nevertheless, as a result of their highly similar morphology, the determination of various species is quite difficult and requires the help of experienced taxonomists. Furthermore, it is very challenging or even impossible to identify nymphal stages or females of some species, e.g., some species of the genus *Sigara* Fabricius, 1775. In term of males of the Corixidae, typical diagnostic traits include the shape and size of the tarsus of the first leg (pala), the arrangement of pala pegs, and the morphology of the genitalia (Jansson, 1986; Savage, 1989). Because aquatic Heteroptera are of high importance for ecological and conservational studies, however, the correct species identification is essential (Hufnagel, Bakonyi & Vásárhelyi, 1999; Whiteman & Sites, 2008; Skern, Zweimüller & Schiemer, 2010). This is especially true for juveniles and females which can, depending on the life history of a species, dominate within a population over a given period of a year (Barahona, Millan & Velasco, 2005; Pfenning & Poethke, 2006; Wachmann, Melber & Deckert, 2006).

In the last few years, new molecular and genomic approaches have become more and more popular to overcome possible drawbacks of this traditional and morphology-based way of species assessment. Given the recent technological advancement of DNA-based methods, in particular in the field of modern high-throughput technologies (Heather & Chain, 2016), it is expected that such techniques will gradually replace traditional field and lab procedures in bioassessment studies over the coming 10–15 years (Leese et al., 2016). For example, the EU COST Action CA15219 on “Developing new genetic tools for bioassessment of aquatic ecosystems in Europe”—or DNAqua-Net (http://dnaqua.net/)—aims to accelerate the use of DNA-based approaches for the monitoring and assessment of aquatic habitats (Leese et al., 2016). Following these considerations, the analysis of single specimens, bulk samples or environmental DNA will be performed routinely as part of modern species diversity assessment studies (Yu et al., 2012; Scheffers et al., 2012; Cristescu, 2014; Shackleton & Rees, 2015; Kress et al., 2015; Creer et al., 2016). However, the effectiveness of all these approaches relies highly on comprehensive sequence libraries that act as valid references (Brandon-Mong et al., 2015; Creer et al., 2016; Morinière et al., 2016). In this context, DNA barcoding represents undoubtedly the most prominent and popular approach using sequence data for valid species identification (Hajibabaei et al., 2007; Miller et al., 2016). The concept of DNA barcoding relies on the postulate that the interspecific genetic variation is higher than the intraspecific variation of the selected marker (Hebert, Ratnasingham & de Waard, 2003; Hebert et al., 2003). As a consequence, every species is characterized by a unique DNA barcode cluster. For animals, an approximately 650 base pair (bp) fragment of the mitochondrial cytochrome *c* oxidase subunit I (COI) gene was proposed as the global standard for the identification of unknown specimens in terms of a given classification (*sensu* Hebert, Ratnasingham & de Waard, 2003; Hebert et al., 2003). However, it should be noted that various problems may affect the use of mitochondrial DNA, e.g., recent speciation events (Balvín et al., 2012; Raupach et al., 2014), heteroplasmy (Boyce, Zwick & Aquadro, 1989; Kavar et al., 2006; Kmiec, Woloszynska & Janska, 2006), incomplete lineage sorting (Petit & Excoffier, 2009), (introgressive) hybridization (Jansson, 1979a, 1979b; Calabrese, 1982; Spence & Wilcox, 1986; Wilcox & Spence, 1986; Savage & Parkin, 1998; Raupach et al., 2014), the presence of alpha-proteobacteria as *Wolbachia* within terrestrial arthropods (Werren, Zhang & Guo, 1995; Xiao et al., 2011; Werren, Baldo & Clark, 2008), and the existence of mitochondrial pseudogenes (Leite, 2012; Song, Moulton & Whiting, 2014). Nevertheless, a vast number of studies across a broad range of different animals demonstrate the efficiency of DNA barcoding (Spelda et al., 2011; Hausmann et al., 2013; Hendrich et al., 2015; Lin, Stur & Ekrem, 2015; Raupach et al., 2015; Barco et al., 2016; Coddington et al., 2016; Morinière et al., 2017).

Despite the fact that more than 45,000 species of true bugs have been described worldwide until now (Henry, 2017), the number of studies analyzing the usefulness of DNA barcodes to discriminate species of this highly diverse insect taxon is still low. Some studies focus on selected species (Rebijith et al., 2012; Zhou et al., 2012; Lis, Lis & Ziaja, 2013), other on specific families (Grebennikov & Heiss, 2014; Kaur & Sharma, 2017), whereas four publications provide a larger representation of various families (Park et al., 2011; Jung, Duwal & Lee, 2011; Raupach et al., 2014; Tembe, Shouche & Ghate, 2014). However, all these studies focused primarily on terrestrial species, analyzing just small number of species belonging to the Gerromorpha and/or Nepomorpha (Park et al., 2011; Jung, Duwal & Lee, 2011; Raupach et al., 2014). To our knowledge, only two publications analyzed aquatic true bugs specifically until now: Castanhole et al. (2013) investigated the variability of 17 barcode sequences of a few species from Brazil, whereas Ebong et al. (2016) successfully tested the usefulness of DNA barcodes to discriminate various species from Cameroon.

The aim of this study was to build-up a baseline for a comprehensive library of DNA barcodes for aquatic Heteroptera (Gerromorpha, Nepomorpha) of Central Europe with a focus on the German fauna and to test the efficiency of DNA barcodes to discriminate the analyzed species. Moreover, our study provides the first thorough molecular study of the aquatic Heteroptera of Germany. In doing so, we analyzed more than 700 DNA barcodes representing more than 60 species. Our library included various morphological similar taxa, e.g., species of the genera *Sigara* Fabricius, 1775 and *Notonecta* Linnaeus, 1758 as well as water striders of the genus *Gerris* Fabricius, 1794 from different localities in Germany. In addition to this we added various specimens from other European countries for comparison, e.g., specimens of the expansive small-bodied backswimmer *Anisops sardeus* Herrich-Schaeffer, 1849 (Soós et al., 2010; Berchi, 2011; Klementová & Svitok, 2014).

## Materials and Methods

### Species collection and identification

**Figure 2:**
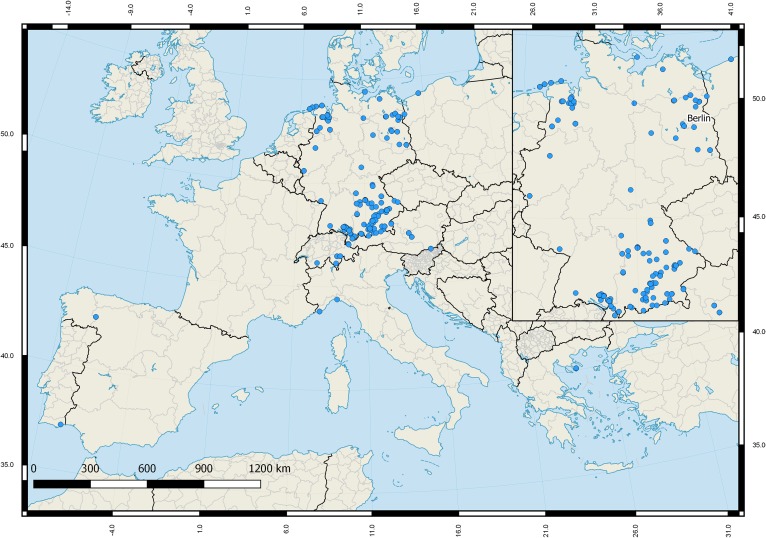
Sampling sites of the studied aquatic true bugs (Gerromorpha, Nepomorpha) across Europe.

All analyzed Gerromorpha and Nepomorpha were collected between the years 2003 and 2017. Most of them were adults (*n* = 584; 96.8%). Specimens were stored in ethanol (96%) immediately after collection and identified by some of the authors (NH, MMG, MJR, PS, RN) using various keys (Nieser, 1982; Jansson, 1986; Savage, 1989; Stoffelen et al., 2013; Strauss & Niedringhaus, 2014) based on the most recent taxonomic classification (Aukema & Rieger, 1995). All specimens were carefully checked multiple times by some of the authors in order to prevent a misidentification. For our analysis we also included 109 DNA barcodes of aquatic bugs that were part of a previous barcoding study of true bugs of Central Europe and in which species identification was verified by the authors for comparison (Raupach et al., 2014). Most of the analyzed bug specimens were collected in Germany (*n* = 616: 86.5%), but various individuals were sampled in Austria (37; 5.2%), Greece (20; 2.8%), Spain (16; 2.3%), Switzerland (8; 1.1%), Italy (7; 1.0%), Poland (6; 0.8%), and Portugal (2; 0.3%) for comparison (Fig. 2). In this context we also included specimens of four species that are not recorded for Germany: I. *Anisops sardeus* Herrich-Schaeffer, 1849 (*n* = 5) from Greece, II. *Mesovelia vittigera* Horváth, 1895 (*n* = 4) from Greece, III. *Sigara dorsalis* (Leach, 1817–1818) (*n* = 1) from Switzerland, and IV. *Velia currens* (Fabricius, 1794) (*n* = 3) from Switzerland. The total data set consisted of 712 DNA barcodes with 63 species that are documented for Germany. Furthermore, the number of analyzed specimens per species ranged from one (eight species) to a maximum of 41 for *Notonecta glauca* Linnaeus, 1758.

### DNA barcode amplification, sequencing, and data depository

The DNA barcode amplification was either performed at the German Centre of Biodiversity Research (Senckenberg am Meer) in Wilhelmshaven, the Carl von Ossietzky University of Oldenburg, or the Bavarian State Collection of Zoology in Munich (SNSB-ZSM). Following the guidelines of DNA barcoding studies (Ratnasingham & Hebert, 2007), all species were documented by photographs before molecular work started. In the majority of the studied animals, all legs of one side of the body were dissected and used for DNA extraction. In case of larger specimens of the genera *Notonecta* Linneaus, 1758, *Ilyocoris* Stål, 1861, *Ranatra* Fabricius, 1790, *Nepa* Linnaeus, 1758, and *Aphelocheirus* Westwood, 1833, however, only one leg was used. For some very small specimens with a body length <3 mm, e.g., species of the genus *Microvelia* Westwood, 1834, complete specimens were used for DNA extraction. All voucher specimens as well as DNA extracts are stored in a local collection at the Carl von Ossietzky University of Oldenburg.

The DNA extraction was performed using the NucleoSpin Tissue Kit by Macherey and Nagel (Düren, Germany), following the extraction protocol. Polymerase chain reaction (PCR) has been used for amplifying the COI barcode fragment by using the established primer pairs LCO1490/HCO2198 (Folmer et al., 1994), LCO1490/NANCY (Simon et al., 1994), jgLCO1490/jgHCO2198 (Geller et al., 2013), or LepF1/LepR1 (Hebert et al., 2004) for most specimens. For various specimens of the Gerromorpha, however, a new specific forward primer HETF1 (5′-ATG AAT TAT TCG AAT TGA AAT AGG-3′) was designed and used in combination with HCO2198 for amplification, resulting in a somewhat smaller fragment with a length of 579 bp of the barcode region. All primers were modified with M13 forward and reverse tails to provide defined base sequences for sequencing (see Ivanova et al., 2007; Khalaji-Pirbalouty & Raupach, 2014).

Barcode amplicons were amplified using illustra™ puReTaq Ready-To-Go PCR Beads (GE Healthcare, Buckinghamshire, UK) in a total volume of 20 μl, containing 17.5 μl sterile molecular grade H_2_O, 2 μl DNA template with an DNA amount between 2 and 150 ng/μl and 0.25 μl of each primer (20 pmol/μl). The PCR thermal conditions included an initial denaturation at 94 °C (5 min), followed by 38 cycles at 94 °C (denaturation, 45 s), 48 °C (annealing, 45 s), 72 °C (extension, 80 s), and a final extension step at 72 °C (7 min). All PCR amplification reactions were conducted using an Eppendorf Mastercycler Pro system (Eppendorf, Hamburg, Germany). Negative and positive controls were included with each round of reactions. Two microliter of the amplified products were verified for size conformity by electrophoresis in a 1% agarose gel with GelRed or SYBR Green using commercial DNA size standards, whereas the remaining PCR product was purified with the NucleoSpin Gel and PCR Clean-up Kit (Macherey-Nagel, Düren, Germany). Purified PCR products were cycle-sequenced and sequenced in both directions at a contract sequencing facility (GATC, Konstanz, Germany) using the given M13 tail sequences. Double stranded sequences became assembled and checked for mitochondrial pseudogenes (numts) analyzing the presence of stop codons, frameshifts as well as double peaks in chromatograms with the Geneious program package version 7.0.4 (Biomatters, Auckland, New Zealand) (Kearse et al., 2012). Ambiguous parts at the 5′-end or 3′-end of the sequences were removed. For verification, BLAST (nBLAST, search set: others, program selection: megablast) and/or BOLD (identification engine; species level barcode records) searches were performed to confirm the identity of all new sequences as bug sequences based on already published sequences.

Detailed voucher information, taxonomic classifications, photos, DNA barcode sequences, used primer pairs and trace files (including their quality) are publicly accessible through the public data set “DS-BAHCE Barcoding Aquatic Heteroptera of Central Europe” (Dataset ID: DOI 10.5883/DS-BAHCE) on the Barcode of Life Data Systems workbench (BOLD; www.boldsystems.org) (Ratnasingham & Hebert, 2007). All new barcode data were also deposited in GenBank (MG665389–MG665993).

### DNA barcode analysis

We analyzed intra- and interspecific distances of the studied aquatic Heteroptera using the provided analytical tools of the BOLD workbench (align sequences: BOLD aligner; ambiguous base/gap handling: pairwise deletion) based on the Kimura 2-parameter model of sequence evolution (K2P; Kimura, 1980). Furthermore, all analyzed COI sequences became subject to the barcode index number (BIN) system implemented in BOLD which clusters DNA barcodes in order to generate operational taxonomic units that closely correspond to species (Ratnasingham & Hebert, 2013). We used a recommended threshold of 2.2% for a rough differentiation of intraspecific as well as interspecific K2P distances (Ratnasingham & Hebert, 2013).

A neighbour-joining cluster analysis (NJ; Saitou & Nei, 1987) was performed for all studied species for a graphical representation of the genetic differences between sequences and clusters of sequences using MEGA v7.0.18 (Kumar, Stecher & Tamura, 2016). The K2P model was chosen as the model for sequence evolution for comparison purposes with previous studies. For validation, non-parametric bootstrap support values were obtained by resampling and analyzing 1,000 replicates (Felsenstein, 1985). All analysis were based on an alignment that was generated using MUSCLE (Edgar, 2004) implemented in MEGA v7.0.18 for all studied barcode sequences. Additionally, statistical maximum parsimony networks were constructed exemplarily for species with interspecific distances ranging from zero to 1% (see Table 1) by using TCS networks (Clement, Posada & Crandall, 2000) as part of the software package of PopArt v.1.7 (Leigh & Bryant, 2015). Such networks allow the identification of haplotype sharing between species as a consequence of recent speciation and/or on-going hybridization processes (Raupach et al., 2010, 2014).

**Table 1:** BOLD distance analysis of the studied Gerromorpha and Nepomorpha.

Family	Species	*n*	PC	BIN	MID	DNN	NNS
Aphelocheiridae	*Aphelocheirus aestivalis*	2	Mono	ABX0398	0	11.86	*Notonecta maculata*
Corixidae	*Arctocorisa carinata*	5	Para	AAJ7903, ACY1261	**2.36**	**1.03**	*Arctocorisa germari*
	*Arctocorisa germari*	1	n. a.	–	0	**1.03**	*Arctocorisa carinata*
	*Callicorixa praeusta*	23	Para	AAK1938	0.31	**0**	*Callicorixa producta*
	*Callicorixa producta*	1	n. a.	AAK1938	0	**0**	*Callicorixa praeusta*
	*Corixa affinis*	13	Mono	ACY0615	1.92	5.92	*Corixa panzeri*
	*Corixa dentipes*	1	n. a.	–	0	6.08	*Corixa punctata*
	*Corixa panzeri*	2	Mono	ACX9506	0	5.92	*Corixa affinis*
	*Corixa punctata*	21	Mono	ACB1799	0.77	6.08	*Corixa dentipes*
	*Cymatia bonsdorffii*	4	Mono	ABX0396	0.62	12.34	*Cymatia coleoptrata*
	*Cymatia coleoptrata*	24	Mono	ACB1796, ADD1561	**9.44**	12.4	*Cymatia bonsdorffii*
	*Cymatia rogenhoferi*	1	n. a.	ACB2132	0	12.7	*Cymatia coleoptrata*
	*Glaenocorisa propinqua*	8	Mono	ABX4248	1.55	9.96	*Sigara semistriata*
	*Hesperocorixa castanea*	14	Mono	ABX0447	0.32	13.22	*Paracorixa concinna*
	*Hesperocorixa linnaei*	8	Mono	ABX0448	0	11.89	*Sigara venusta*
	*Hesperocorixa sahlbergi*	39	Mono	AAN0795	1.7	11.34	*Corixa panzeri*
	*Micronecta griseola*	2	Mono	AAK6480	0	10.63	*Micronecta poweri*
	*Micronecta poweri*	2	Mono	ACB1970	**2.39**	10.63	*Micronecta griseola*
	*Micronecta scholtzi*	6	Mono	AAK6479	0.16	18.58	*Sigara semistriata*
	*Paracorixa concinna*	11	Mono	ABV3365, ADG5371	1.71	7.03	*Sigara semistriata*
	*Sigara distincta*	7	Poly	ABY7152, ABV4484	**5.77**	**0.37**	*Sigara falleni*
	Sigara dorsalis*	1	n. a.	AAJ6688	0	**1.71**	*Sigara striata*
	*Sigara falleni*	12	Poly	AAH9524, ABY7152	**3.37**	**0**	*Sigara iactans*
	*Sigara fossarum*	3	Mono	AAJ6707, ADD1512	**2.82**	2.72	*Sigara scotti*
	*Sigara hellensii*	2	Mono	ADH9592, ACT7694	**4.41**	9.09	*Sigara distincta*
	*Sigara iactans*	12	Poly	ABY7152, AAH9524	**2.67**	**0**	*Sigara falleni*
	*Sigara lateralis*	14	Mono	AAJ6697	0.81	9.84	*Sigara striata*
	*Sigara limitata*	2	Para	ACM1221	0.48	**0.15**	*Sigara semistriata*
	*Sigara nigrolineata*	16	Mono	ACB1978	0.46	10.12	*Sigara semistriata*
	*Sigara scotti*	12	Mono	ACY0807	1.08	2.72	*Sigara fossarum*
	*Sigara semistriata*	5	Poly	ACM1221	0	**0.15**	*Sigara limitata*
	*Sigara stagnalis*	6	Mono	ACY0713	0.55	11.45	*Paracorixa concinna*
	*Sigara striata*	10	Mono	AAJ6688	0.93	**1.71**	*Sigara dorsalis*
	*Sigara venusta*	2	Mono	ABA5309	0	**2.11**	*Sigara semistriata*
Naucoridae	*Ilyocoris cimicoides*	17	Mono	AAF2590	1.03	15.06	*Hesperocorixa sahlbergi*
Nepidae	*Nepa cinerea*	10	Mono	AAK8359	0.34	17.06	*Notonecta maculata*
	*Ranatra linearis*	16	Mono	AAL1328	0.84	15.03	*Notonecta lutea*
Notonectidae	Anisops sardeus*	5	Mono	ABV0079	1.24	12.84	*Notonecta maculata*
	*Notonecta glauca*	41	Mono	AAK4442	1.71	**1.08**	*Notonecta obliqua*
	*Notonecta lutea*	19	Mono	AAN1701	0.68	**1.24**	*Notonecta reuteri*
	*Notonecta maculata*	10	Mono	AAN1703	**2.43**	6.56	*Notonecta glauca*
	*Notonecta obliqua*	9	Mono	AAK4442	0.64	**1.08**	*Notonecta glauca*
	*Notonecta reuteri*	5	Mono	ACE8526	0.46	**1.24**	*Notonecta lutea*
	*Notonecta viridis*	10	Mono	ABV0133	1.18	5.03	*Notonecta glauca*
Pleidae	*Plea minutissima*	17	Mono	ACY0868, AAF3832	**8.3**	10.92	*Notonecta lutea*
Gerridae	*Aquarius najas*	7	Mono	AAN1521	2.14	11.75	*Gerris thoracicus*
	*Aquarius paludum*	19	Mono	AAI7450	1.24	12.61	*Gerris argentatus*
	*Gerris argentatus*	32	Mono	ADD1846	0.72	6.55	*Gerris odontogaster*
	*Gerris asper*	1	n. a.	ABA3327	0	**0.34**	*Gerris lateralis*
	*Gerris costae*	11	Mono	ACI6181	0	7.48	*Gerris thoracicus*
	*Gerris gibbifer*	11	Mono	ACB1756	0.88	8.91	*Gerris lacustris*
	*Gerris lacustris*	38	Mono	ACT3584	1.05	8.91	*Gerris gibbifer*
	*Gerris lateralis*	2	Mono	ABA3327	0.17	**0.34**	*Gerris asper*
	*Gerris odontogaster*	19	Mono	ABU6679, ADD1838	1.59	6.55	*Gerris argentatus*
	*Gerris thoracicus*	6	Mono	ACB1745	0.35	7.48	*Gerris costae*
	*Limnoporus rufoscutellatus*	3	Mono	AAV0261	0.88	11.86	*Gerris asper*
Hebridae	*Hebrus pusillus*	2	Mono	AAN0981	0.15	14.32	*Hebrus ruficeps*
	*Hebrus ruficeps*	7	Mono	AAI6967	0.15	14.32	*Hebrus pusillus*
Hydrometridae	*Hydrometra gracilenta*	9	Mono	AAN0857	0.46	13.06	*Hydrometra stagnorum*
	*Hydrometra stagnorum*	21	Mono	AAK5632	0.62	13.06	*Hydrometra gracilenta*
Mesoveliidae	*Mesovelia furcata*	17	Mono	AAN2451	1.39	16.24	*Mesovelia vittigera*
	Mesovelia vittigera*	4	Mono	ACD4048	**2.32**	16.24	*Mesovelia furcata*
Veliidae	*Microvelia buenoi*	1	n. a.	ACY1789	0	15.06	*Gerris costae*
	*Microvelia reticulata*	27	Mono	AAG4341	0.77	15.04	*Gerris asper*
	*Velia caprai*	20	Mono	AAN0455	1.1	4.94	*Velia saulii*
	Velia currens*	3	Mono	ADI1962	0	2.82	*Velia saulii*
	*Velia saulii*	1	n. a.	ABX0836	0	2.82	*Velia currens*

Notes:

With the number of analyzed specimens (*n*), phylogenetic categories (PC), barcode index number (BIN), maximum intraspecific pairwise K2P distances (MID), minimum interspecific pairwise K2P distances to the nearest neighbour species (DNN), and the nearest neighbour species (NNS). Maximum intraspecific distances >2.2% and minimum interspecific distances <2.2% are marked in bold. At least one specimen of the compared species showed a distance value above or below this threshold in terms of a pairwise comparison. Asterisks (*) indicate species not recorded for Germany.

## Results

Our analyzed DNA barcode library comprised 63 species that are documented for Germany, representing 91% of the known aquatic bug species diversity of this country (Nepomorpha: *n* = 43 (92%); Gerromorpha: *n* = 20 (91%)), and additional four species that were collected in other countries and not recorded for Germany. In total, we generated 603 new barcodes of 64 species. The complete alignment of all analyzed sequences (*n* = 712) had a length of 658 bp, with fragments lengths ranging from a minimum of 366 bp to the full barcode fragment size of 658 bp. For some studied specimens of *Cymatia coleoptrata* (Fabricius, 1777) (*n* = 22), our analysis revealed two characteristic deletions of 39 (alignment position: 110–148) (Fig. S1) and nine nucleotides (629–637) for all studied specimens. Average base frequencies were *A* = 32%, *C* = 17%, *G* = 16%, and *T* = 35%. For eight species only one barcode sequence was generated (Table 1). Intraspecific distances ranged from zero to maximum values of 8.3% (*Plea minutissima* Leach, 1817) and 9.44% (*C. coleoptrata*) (Table 1). Maximum intraspecific pairwise distances with values >2.2% were found for 11 species (Table 1). In terms of interspecific divergence, values ranged from zero to 18.58%, with 18 species pairs having values <2.2% (Table 1). We found interspecific distances below 1% for nine species. Unique BINs were recorded for 55 species, whereas two BINs were identified for 10 species (Table 1). For two species that were represented only by one specimen, namely *Arctocorisa germari* (Fieber, 1848) and *Corixa dentipes* Thomson, 1869, our sequences did not have he required fragment length of at least 400 bp to fulfill the criteria for BIN assignment. As consequence, no BINs were available for these two species.

**Figure 3:**
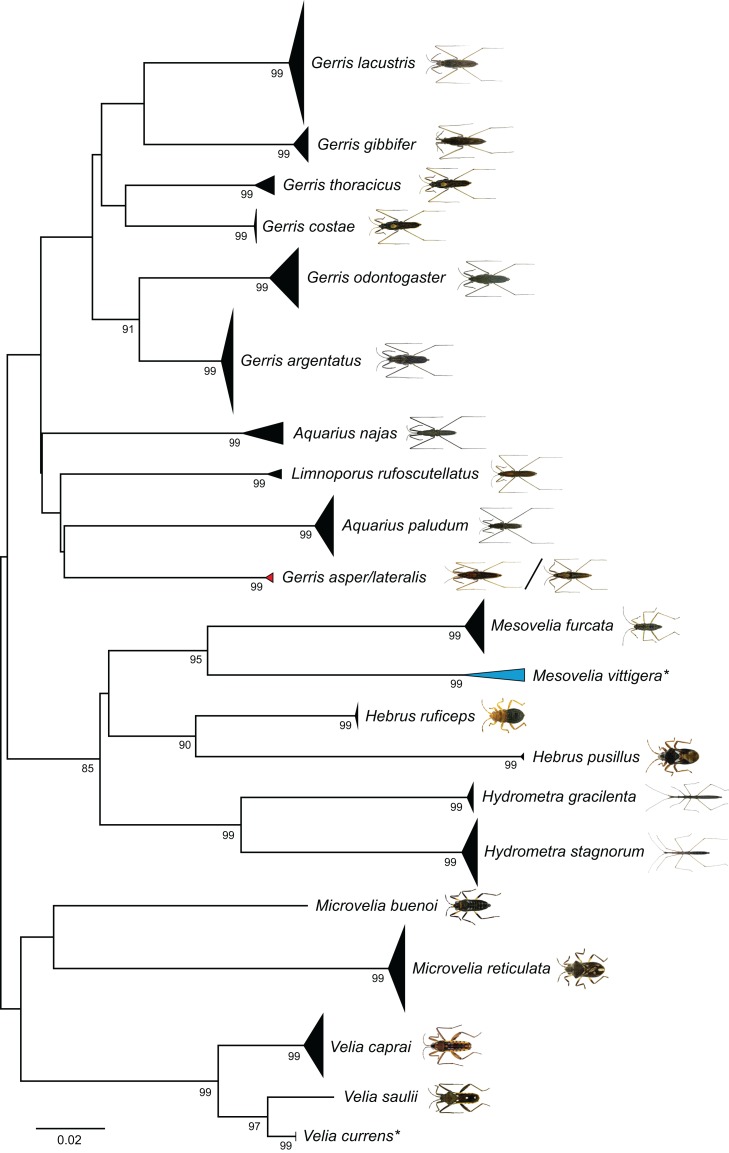
Neighbour-joining (NJ) topology of the analyzed species of the Gerromorpha based on Kimura 2-parameter distance. Triangles indicate the relative number of individual’s sampled (height) and sequence divergence (width). Blue triangles indicate species with intraspecific maximum pairwise distances >2.2%, red triangles species pairs with interspecific distances <2.2%. Numbers next to nodes represent non-parametric bootstrap values >80% (1,000 replicates). Asterisks indicate species not recorded in Germany. All images were obtained from http://www.corisa.de.

**Figure 4:**
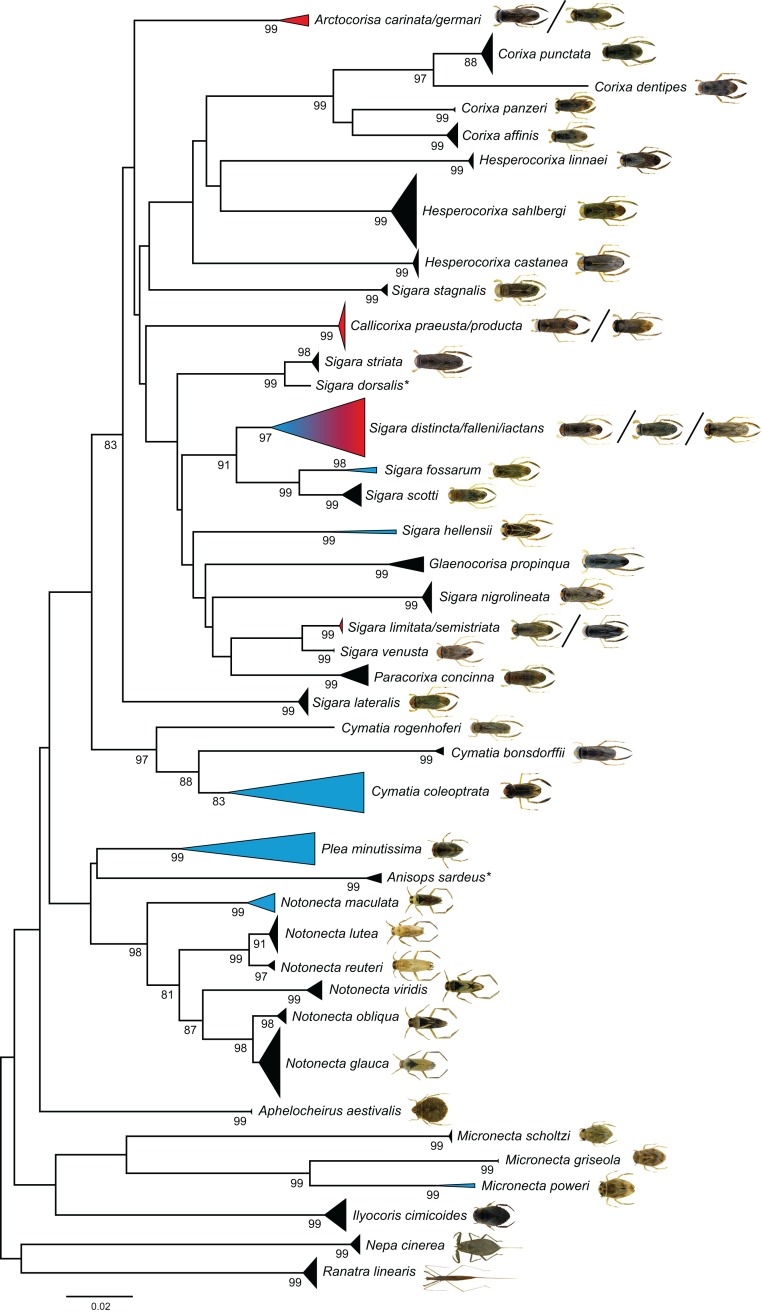
Neighbour-joining (NJ) topology of the analyzed species of the Nepomorpha based on Kimura 2-parameter distance. Triangles indicate the relative number of individual’s sampled (height) and sequence divergence (width). Blue triangles indicate species with intraspecific maximum pairwise distances >2.2%, red triangles species with interspecific distances <2.2%. Numbers next to nodes represent non-parametric bootstrap values ≥80% (1,000 replicates). Asterisks indicate species not recorded in Germany. All images were obtained from http://www.corisa.de.

Our NJ analysis based on K2P distances revealed two large and distinct clusters, separating all analyzed Gerromorpha and all Nepomorpha specimens from each other (Fig. S2). For a better presentation, the topology has been split on this basis and shown in two figures (Gerromorpha: Fig. 3, Nepomorpha: Fig. 4). We found non-overlapping clusters with bootstrap values >90% for 57 species (85%) (Figs. 3 and 4). Of the analyzed 59 species with more than one specimen, 52 (88%) were monophyletic, three (5%) paraphyletic, and four (7%) polyphyletic (Table 1; Fig. S2).

**Figure 5:**
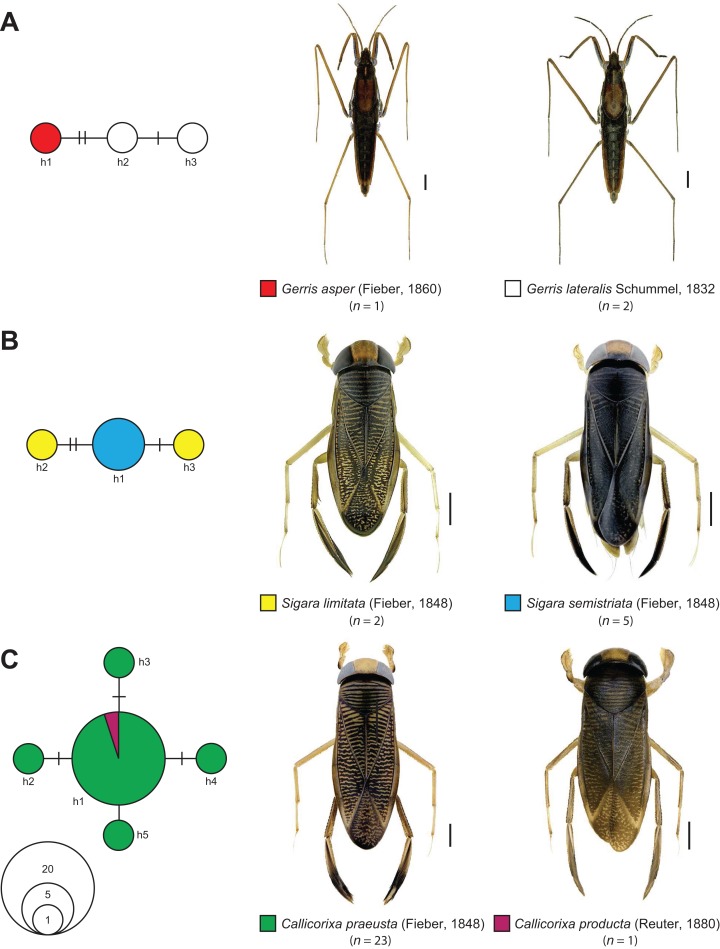
Maximum statistical parsimony network of various species of the Gerromorpha and Nepomorpha with interspecific K2P-based distances of COI sequences <1%. (A) *Gerris asper* (Fieber, 1860) (*n* = 1) and *Gerris lateralis* Schummel, 1832 (*n* = 2); (B) *Sigara limitata* (Fieber, 1848) (*n* = 2) and *Sigara semistriata* (Fieber, 1848) (*n* = 5); (C) *Callicorixa praeusta* (Fieber, 1848) (*n* = 23) and *Callicorixa producta* (Reuter, 1880) (*n* = 1). Used settings included default settings for connection steps whereas gaps were treated as fifth state. Each line represents a single mutational change whereas small black dots and small black lines indicate missing haplotypes. The diameter of the circles is proportional to the number of haplotypes sampled (see open half circles with numbers). Color codes were given for each species. Scale bars = 1 mm. Aquatic bug images were obtained from http://www.corisa.de.

**Figure 6:**
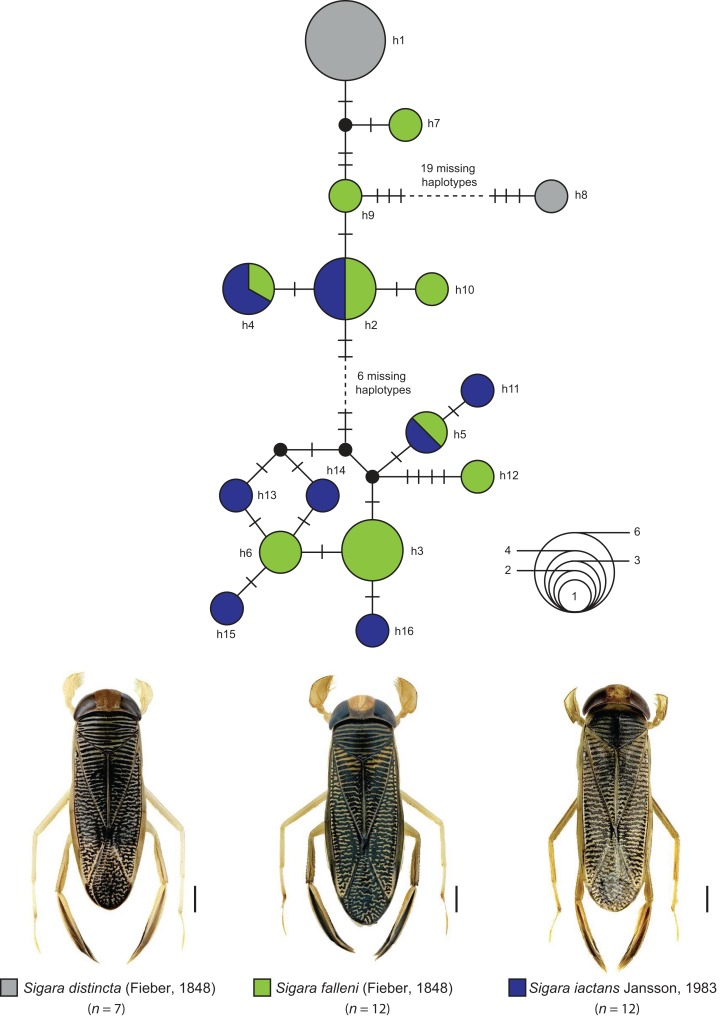
Maximum statistical parsimony network of three *Sigara* species with interspecific K2P-based distances of COI sequences <1%. Used settings included default settings for connection steps whereas gaps were treated as fifth state. Each line represents a single mutational change whereas small black dots and small black lines indicate missing haplotypes. The diameter of the circles is proportional to the number of haplotypes sampled (see open half circles with numbers). Color codes were given for each species. Scale bars = 1 mm. Aquatic bug images were obtained from http://www.corisa.de.

The statistical maximum parsimony network analysis of species with interspecific distances below 1% revealed a close relationship between *Gerris asper* (Fieber, 1860) (*n* = 1) and *Gerris lateralis* Schummel, 1832 (*n* = 2) (Fig. 5). We found three haplotypes with a frequency of one (singletons) that were separated by only one or two mutational steps, with haplotype h1 (*G. asper*) connected with h2 (*G. lateralis*), which was in turn connected with haplotype h3 (*G. lateralis*). A similar situation was observed for *Sigara limitata* (Fieber, 1848) (*n* = 2) and *Sigara semistriata* (Fieber, 1848) (*n* = 5) (Fig. 5). Three different haplotypes were identified, with h1 representing all studied specimens of *S. semistriata*. Both unique haplotypes of *S. limitata* (h2, h3) were directly connected to this haplotype by two or three mutational steps. In the case of *Callicorixa praeusta* (Fieber, 1848) (*n* = 23) and *Callicorixa producta* (Reuter, 1880) (*n* = 1) we found five different haplotypes (Fig. 5), with h1 representing the dominant haplotype which includes 19 specimens of *C. praeusta* and the only specimen of *C. producta*. All other four haplotypes (h2–h5) were only scored in one specimen and connected with h1 by one or two mutational steps. A much more complex network was revealed for *Sigara distincta* (Fieber, 1848) (*n* = 7), *Sigara falleni* (Fieber, 1848) (*n* = 12), and *Sigara iactans* Jansson, 1983 (*n* = 12) (Fig. 6). We identified 16 different haplotypes in total, with six haplotypes (h1–h6) shared by more than one specimen. Three of these haplotypes (h2, h3, h5) were shared by specimens of *S. falleni* and *S. iactans*. Furthermore, haplotypes of both previously mentioned species were randomly distributed within the network. In many cases, haplotypes of *S. falleni* were separated merely by two mutational steps from haplotypes of *S. iactans* (e.g., h6 and h13) and vice versa. We found four singletons for *S. falleni* and five for *S. iactans*. In contrast to these two species, we identified only two haplotypes (h1, h8) for the seven analyzed specimens of *S. distincta*. Moreover, most specimens (*n* = 6) were identical (h1) and located at the periphery of the network. The other haplotype (h8), a singleton collected among others at Apen (Lower Saxony), was separated by more than 25 mutational steps from the network and represents the most isolated haplotype in this network by far. Therefore, *S. distincta* shared no haplotypes with other species.

## Discussion

Our comprehensive DNA barcode library represents an important step for the molecular characterization of the freshwater fauna in Central Europe and adjacent regions. As COI sequences are used routinely in phylogeographic, phylogenetic, and evolutionary studies as well, our data can be also implemented in projects analyzing the genetic variation of species in relation to historical, geographical, and ecological factors (Galacatos, Cognato & Sperling, 2002; Damgaard, 2005, 2008b; Gagnon & Turgeon, 2010; Ye et al., 2016). Unique BINs were found for 55 species, allowing a valid identification of 82% of the analyzed 67 species. Distinct and monophyletic lineages were revealed for 52 species (78%). Our study also indicates the need of further detailed taxonomic revisions, using state-of-the-art methods for a fine-scaled characterization (Raupach et al., 2016). This is especially true for the species-rich family Corixidae. In the following we will discuss noticeable species with high intraspecific and/or low interspecific distances more in detail.

### Interspecific K2P distances with values below 2.2%

The efficiency of DNA barcoding highly depends on distinct mitochondrial lineages, ideally coupled with moderate to high genetic interspecific distances. If sister species, however, have low interspecific distances and haplotype sharing as a result of a recent ancestry and/or ongoing gene flow, DNA barcoding will fail (Tautz et al., 2003; Frezal & Leblois, 2008; Raupach & Radulovici, 2015). For the analyzed species of the Gerromorpha and Nepomorpha, minimum interspecific K2P distances with values below 2.2% were found for 18 species (Table 1). Distance values ranged from 0% (four species: *C. praeusta* (Fieber, 1848), *C. producta* (Reuter, 1880), *S. falleni* (Fieber, 1848), *Sigara iactans* Jansson, 1983) to 2.11% (*Sigara venusta* (Douglas & Scott, 1869)). Distinct monophyletic clusters, however, were revealed for *Notonecta obliqua* Thunberg, 1787 and *Notonecta glauca* Linnaeus, 1758 (1.08%), *Notonecta lutea* Müller, 1776 and *Notonecta reuteri* Hungerford, 1928 (1.24%), *Sigara dorsalis* (Leach, 1817) and *Sigara striata* (Linnaeus, 1758) (1.71%) (but see Savage & Parkin, 1998), and *S. venusta* (Douglas & Scott, 1869) and *S. limitata* (Fieber, 1848)/*S. semistriata* (Fieber, 1848) (2.11%), indicating a close relationship of these species pairs with distinct lineages (Table 1). Furthermore, the analyzed specimen of *A. germari* (Fieber, 1848) was nested in the paraphyletic cluster of *Arctocorisa carinata* (Sahlberg, 1819) (1.03%) (Fig. S1). In this context it should be noted that experimental crosses gave viable hybrids between both *Arctocorisa* species with intermediate characters (Jansson, 1979a). These examples show that recent speciation events as well as hybridization may represent important processes in these groups. Future studies including more specimens and other genetic markers should be conducted to resolve the eco-evolutionary events leading to the low interspecific variation. Species pairs with interspecific K2P distances <1% will be discussed more in detail below.

### Species pairs with interspecific distances below 1%

#### *Gerris asper* (Fieber, 1860) and *Gerris lateralis* Schummel, 1832

From a morphological perspective, both species are very similar (Wagner & Zimmermann, 1955; Schuster, 1983; Wachmann, Melber & Deckert, 2006). Not surprisingly, *G. asper* is suggested as a south-eastern vicariant of its boreo-montane sister species *G. lateralis* (Jeziorski et al., 2012). Whereas *G. lateralis* has a distribution ranging from Europe to the Far East of Russia, *G. asper* is found in Southern and Central Europe, extending to Afghanistan (Jeziorski et al., 2012). In spite of the fact that our sample sizes were very small (*G. asper*: *n* = 1, *G. lateralis*: *n* = 2), our molecular data set clearly support the proposed close relationship of both water striders species (Fig. 5; Table 1). Future studies including more specimens covering a larger geographic range are needed to test whether both taxa represent distinct lineages or hybridization still takes place as it is known from other species of this genus (Calabrese, 1982).

#### Sigara limitata (Fieber, 1848) and Sigara semistriata (Fieber, 1848)

Both species belong to the subgenus *Retrocorixa* Walton, 1940 and have a similar distribution, ranging from Europe eastwards to Siberia (Jansson, 1986; Wachmann, Melber & Deckert, 2006; Coulianos, Økland & Økland, 2008). A close relationship as it has been indicated by our data has not been proposed yet. In contrast to our results, morphological characters suggest *S. venusta* (Douglas & Scott, 1869) as sister species of *S. semistriata* (see Jansson, 1986). As part of our study, *S. venusta* represents the sister species of *S. limitata* and *S. semistriata* with a distance of 2.11% (Fig. 5; Table 1). Due to the fact that neither *S. limitata* nor *S. semistriata* were monophyletic and the observed interspecific distances were very low (0.15%) (Table 1), we suggest a recent ancestry of both species. Hybrids are currently not known. Future studies are needed to verify this hypothesis.

#### Callicorixa praeusta (Fieber, 1848) and Callicorixa producta (Reuter, 1880)

The genus *Callicorixa* White, 1873 includes five medium sized species (6–8 mm) that are recorded for Europe, with two species documented in Central Europe. Specimens of *C. praeusta* can be found throughout most Europe except the Mediterranean region reaching to the Far East of Russia, whereas the distribution of *C. producta* ranges from the Northern parts of Central Europe to Fennoscandia, Northern Russia, Kazakhstan, Mongolia, and Siberia (Jansson, 1986; Wachmann, Melber & Deckert, 2006; Coulianos, Økland & Økland, 2008). Most identification keys for this genus rely largely on the shape and intensity of dark areas of the hind tarsus 1 (Jansson, 1986; Savage, 1989; Strauss & Niedringhaus, 2014). While this morphological trait is fairly good for the determination of most typical specimens, existing variation is rather wide, making it unreliable in many cases (Jansson, 1986). Similar to other species, our DNA barcode data give evidence for a recent ancestry or ongoing gene flow between *C. praeusta* and *C. producta* (Fig. 5). However, only one (female) specimen of *C. producta* was available, demonstrating the need for more detailed studies to clarify the underlying processes.

#### Sigara distincta (Fieber, 1848), Sigara falleni (Fieber, 1848), and Sigara iactans Jansson, 1983

Some decades ago, a comprehensive revision revealed that the well-known species *S. falleni* of the subgenus *Subsigara* Stichel, 1935 was actually a mixture of four closely related and highly similar species, including *S. iactans* (see Jansson, 1983). Whereas the identification of females is not always reliable, males of both species can be recognized by the shape of their pala: specimens of *S. falleni* are characterized by triangular pala, whereas trapezoidal pala are found for *S. iactans* (Jansson, 1983, 1986). Intermediate specimens, however, have been also documented and indicate on-going hybridization between both species (Jansson, 1983, 1986).

Water bugs of *S. distincta* are found from the British Isles through North and Central Europe to Asia as far as East Siberia and Mongolia (Jansson, 1986; Savage, 1989; Coulianos, Økland & Økland, 2008). A similar distribution is known for *S. falleni*, ranging throughout most of Europe eastwards to Siberia and China (Jansson, 1986; Savage, 1989; Coulianos, Økland & Økland, 2008). In contrast to both previous species, *S. iactans* is found in two disjunct areas, one in North and Central Europe, and the other in Southeastern Europe (Jansson, 1986; Wachmann, Melber & Deckert, 2006). Our DNA barcode data revealed multiple haplotype sharing between *S. falleni* and *S. iactans*, supporting the close relationship and on-going hybridization between both species (Fig. 6). Beside this, our results revealed a close relationship of *Sigara (Subsigara) distincta* with *S. falleni* and *S. iactans*, as it has been discussed in the past also (Jansson, 1986). However, we found no shared haplotypes yet. Additional studies involving more specimens of a larger geographic region are needed to validate the species status within this subgenus.

### Intraspecific K2P distances with values >2.2%

Various phenomena can generate distinct lineages within DNA barcode data, e.g., phylogeographic processes (Andersen et al., 2000; Damgaard, 2005, 2008b; Ye et al., 2016), the presence of maternally inherited endosymbionts as *Wolbachia* (Lis, Maryańska-Nadachowska & Kajtoch, 2015), or the existence of cryptic species (Paterson et al., 2016; Jiu et al., 2017). In this context we found 11 species with intraspecific K2P distances >2.2%, ranging from 2.32% (*Mesovelia vittigera* Horváth, 1895) to a maximum of 9.44 (*C. coleoptrata* (Fabricius, 1777)). For most species, excluding *S. iactans* (2.67%), *S. falleni* (3.37%), and *S. distincta* (5.77%) (see Discussion above), we are currently unable to clarify the background of the observed high nucleotide distances and distinct lineages based on the given data set. However, exceptionally high intraspecific distances with values >8% were found within the pygmy backswimmer *Plea minutissima* Leach, 1817 (8.3%) and *C. coleoptrata* (Fabricius, 1777) (9.44%) (Table 1). Both will be discussed more in detail.

### Small and cryptic: two highly distinct DNA barcode clusters within *Plea minutissima* Leach, 1817

**Figure 7:**
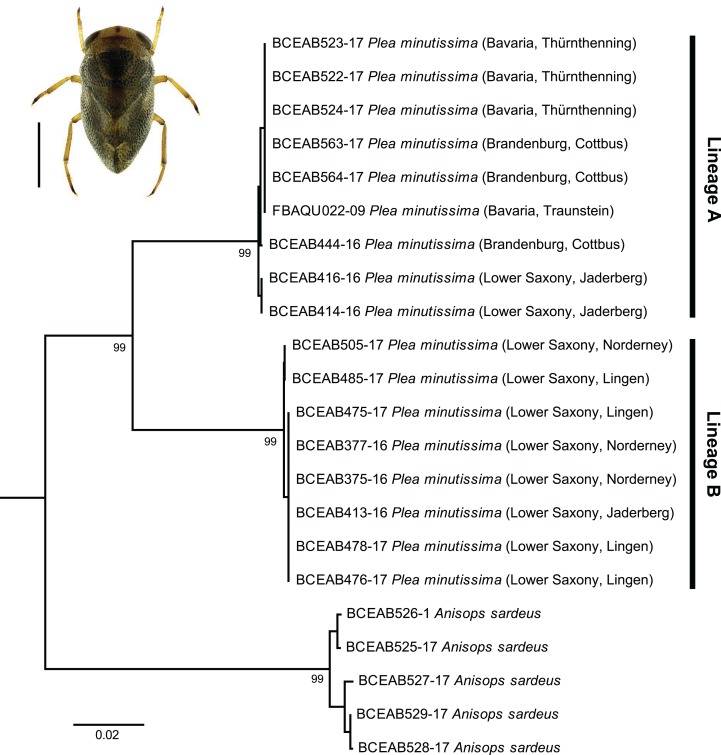
Subtree of the neighbour-joining topology of the analyzed specimens of *Plea minutissima* Leach, 1817. Branches with specimen ID-Number from BOLD and species names. Numbers next to internal branches are non-parametric bootstrap values (in %). Scale bar = 1 mm. Image obtained from http://www.corisa.de.

Pygmy backswimmers are small bugs, usually less than 3.5 mm in length and confine themselves to the vegetation in which they hide and where they prey on mosquito larvae and other small arthropods (Schuh & Slater, 1995). For Europe, only one species of the Pleidae is recorded, namely *P. minutissima*. As part of our study we found two distinct lineages within the 16 analyzed specimens with high distances ranging from 8.1% to 8.3%. Both lineages were supported by high bootstrap values (99%) (Fig. 7). Most specimens of lineage A (*n* = 8) were found in Brandenburg and Bavaria, but also two specimens were collected in Lower Saxony (Jaderberg). In contrast to this, all specimens of lineage B (*n* = 8) were collected in Lower Saxony (Jaderberg, Lingen, Norderney). Whether this surprisingly high molecular diversity is a result of effects as incomplete lineage sorting (Damgaard, 2008a) or whether we found evidence for the existence of two sibling species (Damgaard, 2005), is not within the scope of this study but clearly needs further investigation.

### A currently unknown species of the genus *Cymatia* Flor, 1860?

**Figure 8:**
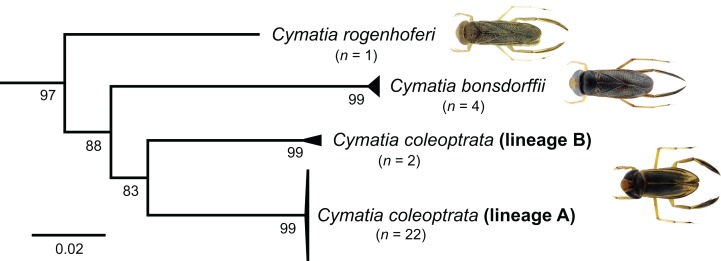
Subtree of the neighbour-joining topology of the analyzed species of the genus *Cymatia* Flor, 1860. Numbers next to internal branches are non-parametric bootstrap values (in %). Images obtained from http://www.corisa.de.

For the genus *Cymatia*, three European species are documented so far: *C. coleoptrata* (Fabricius, 1777), *C. bonsdorffii* (Sahlberg, 1819), and *C. rogenhoferi* (Fieber, 1864). In terms of a morphological identification, all species can be identified according to their size and hemelytral patterns without doubt (Jansson, 1986, Stoffelen et al., 2013). Our study revealed two distinct lineages within the analyzed specimens of *C. coleoptrata* (lineage A and B), with a K2P distances ranging from 9.13% to 9.42% and bootstrap support values of 99% (Fig. 8). Whereas lineage A includes 22 specimens from Lower Saxony (*n* = 21, Lingen) and Baden-Württemberg (*n* = 1, Wolperstwende), lineage B contains two specimens that were collected in Brandenburg (Voßberg). Both specimens of lineage B were small adult males with a body size between 4.3 and 4.5 mm and were identified using morphological traits as *C. coleoptrata* at first sight. Interestingly, their barcode sequences did not have the characteristic nucleotide deletions of this species (Fig. S1). Furthermore, we found no other similar sequences using the BOLD identification engine (Best ID: *C. coleoptrata*) (date of request: 2017-11-20). Unfortunately, both *Cymatia* vouchers were lost, preventing a closer reanalysis of the specimens. Nevertheless, our results should motivate heteropterologists to study more specimens of this genus, in particular from the Eastern parts of Germany, in order to verify the presence of this putative new species.

## Conclusion

In our study we lay the foundations for a comprehensive DNA barcode data set for the aquatic Heteroptera in Central Europe and adjacent regions, which will act as useful reference library for freshwater bioassessment studies using modern high-throughput sequencing technologies. Unique BINs were revealed for 55 species, representing 82% of the analyzed 67 species. Furthermore, monophyletic lineages were found for 52 species (78%). Nevertheless, our molecular data highlights discordance between the generally accepted but exclusively morphologically based taxonomy and observed molecular diversity within some species of the Gerromorpha and Nepomorpha. The analysis of additional specimens from other localities and of other molecular markers, e.g., microsatellites or SNPs, will give us more insights into the taxonomic status of these species as well as in the eco-evolutionary processes underlying the observed genetic patterns. However, it should be kept in mind that the traditional aims of taxonomy are unchanged and include various aspects, e.g., detailed high-quality descriptions and delimitation of species, a classification that reflects evolution, a dynamic nomenclature, and fast and reliable identification tools. Therefore, our DNA barcode library may be considered as a promoter for such studies.

## Supplemental Information

10.7717/peerj.4577/supp-1 Supplemental Information 1 Aligned DNA barcodes of the data set DS-BAHCE Barcoding Aquatic Heteroptera of Central Europe. Accession numbers are requested and will be added in proof.

10.7717/peerj.4577/supp-2 Supplemental Information 2 Screenshot of the complete alignment of all studied aquatic Heteroptera (Gerromorpha, Nepomorpha) showing a gap of 39 nucleotides (13 amino acids) within the analyzed specimens of *Cymatia coleoptrata* (Fabricius, 1777) from position 110 to 148. Amino acid classification accords to the IUPAC-IUB single-letter amino acid codes. Visualization was performed using the Geneious program package version 7.0.4.

10.7717/peerj.4577/supp-3 Supplemental Information 3 Neighbor Joining topology of all analyzed aquatic bug specimens based on Kimura 2-parameter distances. Specimens are classified using ID numbers from BOLD and species name. Numbers next to nodes represent non-parametric boot-strap values (1,000 replicates, in %).

10.7717/peerj.4577/supp-4 Supplemental Information 4 Supplemental information.

## Additional Information and Declarations

### DNA Deposition

The following information was supplied regarding the deposition of DNA sequences: Detailed information are publicly accessible and can be found via the dataset ID of BOLD: DOI 10.5883/DS-BAHCE.

### Data Availability

The following information was supplied regarding data availability: The aligned DNA barcodes of the analyzed data set DS-BAHCE Barcoding Aquatic Heteroptera of Central Europe are provided in Supplemental Dataset File.
