# Supplementary figures and images for: From water striders to water bugs: the molecular diversity of aquatic Heteroptera (Gerromorpha, Nepomorpha) of Germany based on DNA barcodes
Source: PeerJ. 2018 May 2;6:e4577. doi: 10.7717/peerj.4577 (PMC5936072; doi:10.7717/peerj.4577)

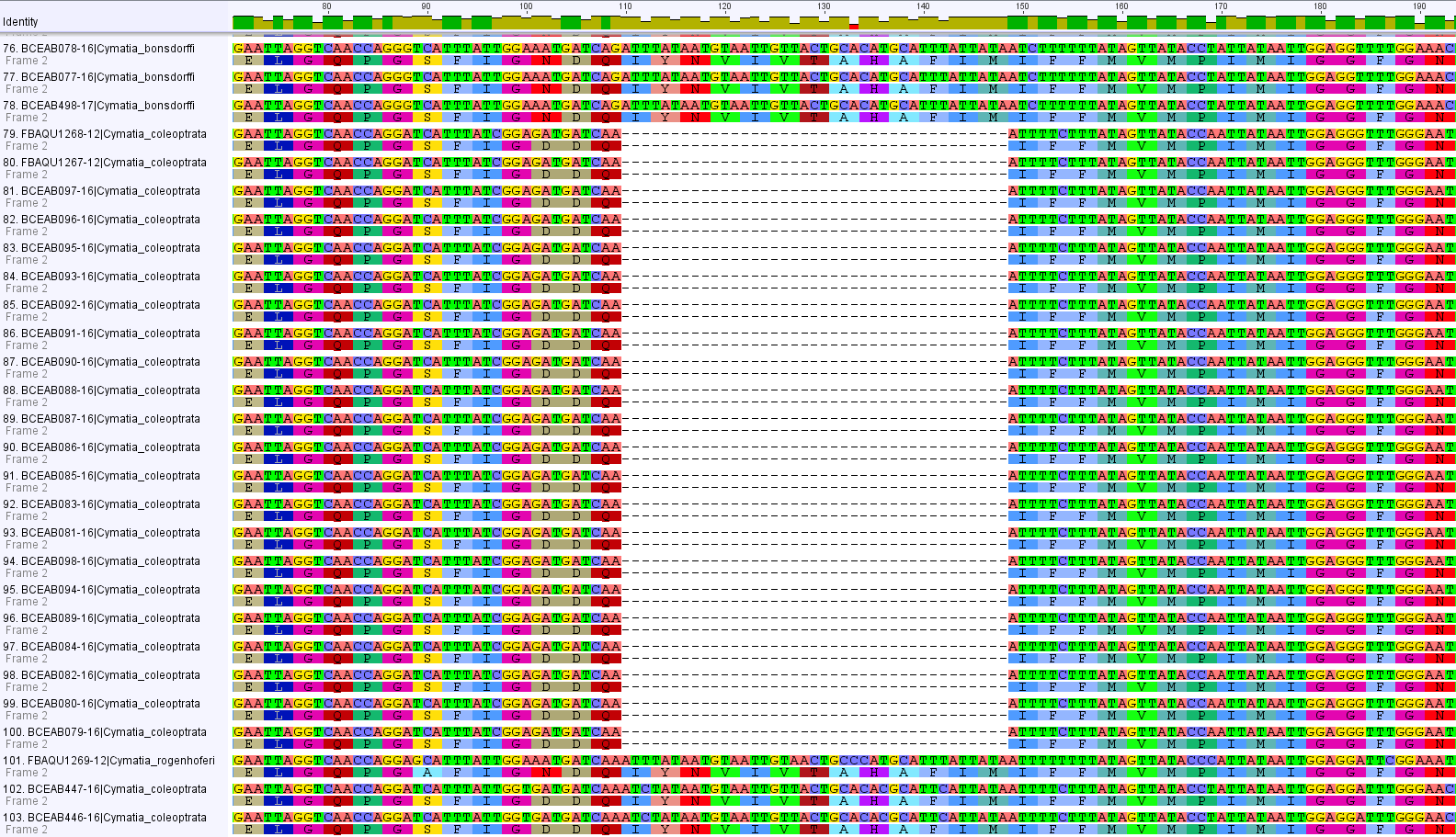

Supplement: Supplemental Information 2 — Amino acid classification accords to the IUPAC-IUB single-letter amino acid codes. Visualization was performed using the Geneious program package version 7.0.4. [file peerj-06-4577-s002.png]
