# Supplementary material for: From water striders to water bugs: the molecular diversity of aquatic Heteroptera (Gerromorpha, Nepomorpha) of Germany based on DNA barcodes
Source: PeerJ. 2018 May 2;6:e4577. doi: 10.7717/peerj.4577 (PMC5936072; doi:10.7717/peerj.4577)
Supplement: Supplemental Information 3 — Specimens are classified using ID numbers from BOLD and species name. Numbers next to nodes represent non-parametric boot-strap values (1,000 replicates, in %). [file peerj-06-4577-s003.pdf]

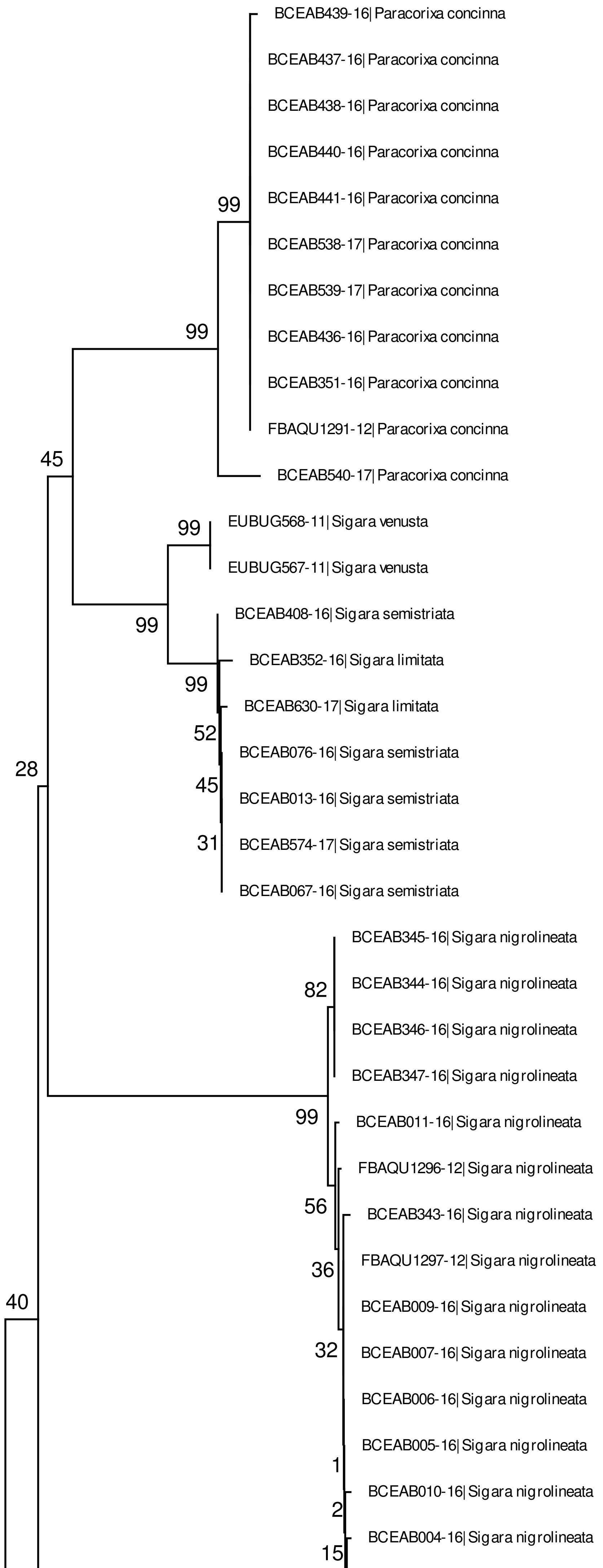
BCEAB439-16| Paracorixa concinna
BCEAB437-16| Paracorixa concinna
BCEAB438-16| Paracorixa concinna
BCEAB440-16| Paracorixa concinna
99
BCEAB441-16| Paracorixa concinna
BCEAB538-17| Paracorixa concinna
BCEAB539-17| Paracorixa concinna
99
BCEAB436-16| Paracorixa concinna
BCEAB351-16| Paracorixa concinna
FBAQU1291-12| Paracorixa concinna
45
BCEAB540-17| Paracorixa concinna
99
EUBUG568-11| Sigara venusta
EUBUG567-11| Sigara venusta
BCEAB408-16| Sigara semistriata
99
BCEAB352-16| Sigara limitata
99
BCEAB630-17| Sigara limitata
52
BCEAB076-16| Sigara semistriata
28
45
BCEAB013-16| Sigara semistriata
31
BCEAB574-17| Sigara semistriata
BCEAB067-16| Sigara semistriata
BCEAB345-16| Sigara nigrolineata
82
BCEAB344-16| Sigara nigrolineata
BCEAB346-16| Sigara nigrolineata
BCEAB347-16| Sigara nigrolineata
99
BCEAB011-16| Sigara nigrolineata
FBAQU1296-12| Sigara nigrolineata
56
BCEAB343-16| Sigara nigrolineata
36
FBAQU1297-12| Sigara nigrolineata
40
BCEAB009-16| Sigara nigrolineata
32
BCEAB007-16| Sigara nigrolineata
BCEAB006-16| Sigara nigrolineata
BCEAB005-16| Sigara nigrolineata
1
BCEAB010-16| Sigara nigrolineata
2
BCEAB004-16| Sigara nigrolineata
15

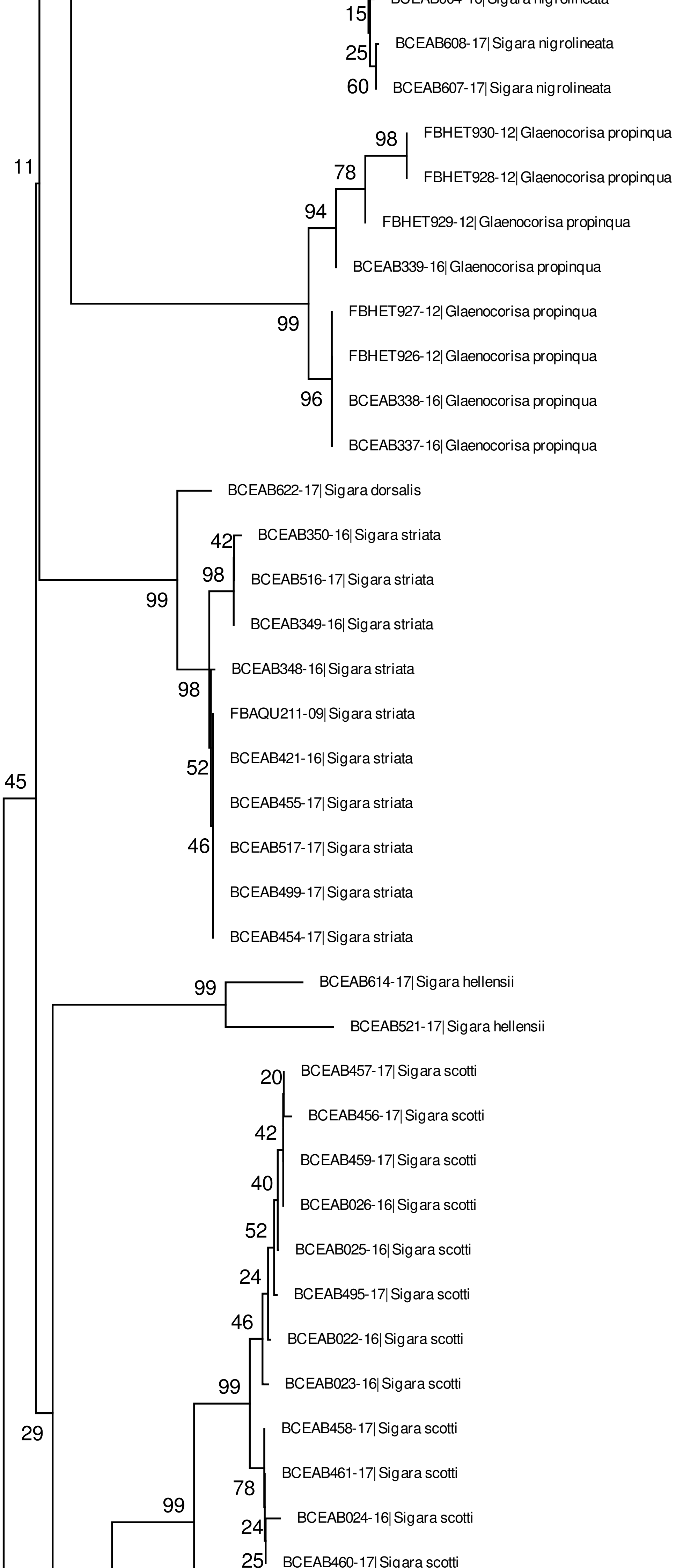
15
25
BCEAB608-17| Sigara nigrolineata
60
BCEAB607-17| Sigara nigrolineata
98
FBHET930-12| Glaenocorisa propinqua
78
FBHET928-12| Glaenocorisa propinqua
94
FBHET929-12| Glaenocorisa propinqua
11
BCEAB339-16| Glaenocorisa propinqua
FBHET927-12| Glaenocorisa propinqua
99
FBHET926-12| Glaenocorisa propinqua
96
BCEAB338-16| Glaenocorisa propinqua
BCEAB337-16| Glaenocorisa propinqua
BCEAB622-17| Sigara dorsalis
42
BCEAB350-16| Sigara striata
98
BCEAB516-17| Sigara striata
99
BCEAB349-16| Sigara striata
BCEAB348-16| Sigara striata
98
FBAQU211-09| Sigara striata
52
BCEAB421-16| Sigara striata
45
BCEAB455-17| Sigara striata
46
BCEAB517-17| Sigara striata
BCEAB499-17| Sigara striata
BCEAB454-17| Sigara striata
99
BCEAB614-17| Sigara hellensii
BCEAB521-17| Sigara hellensii
20
BCEAB457-17| Sigara scotti
BCEAB456-17| Sigara scotti
42
BCEAB459-17| Sigara scotti
40
BCEAB026-16| Sigara scotti
52
BCEAB025-16| Sigara scotti
24
BCEAB495-17| Sigara scotti
46
BCEAB022-16| Sigara scotti
99
BCEAB023-16| Sigara scotti
29
BCEAB458-17| Sigara scotti
78
BCEAB461-17| Sigara scotti
99
24
BCEAB024-16| Sigara scotti
25
BCEAB460-17| Sigara scotti

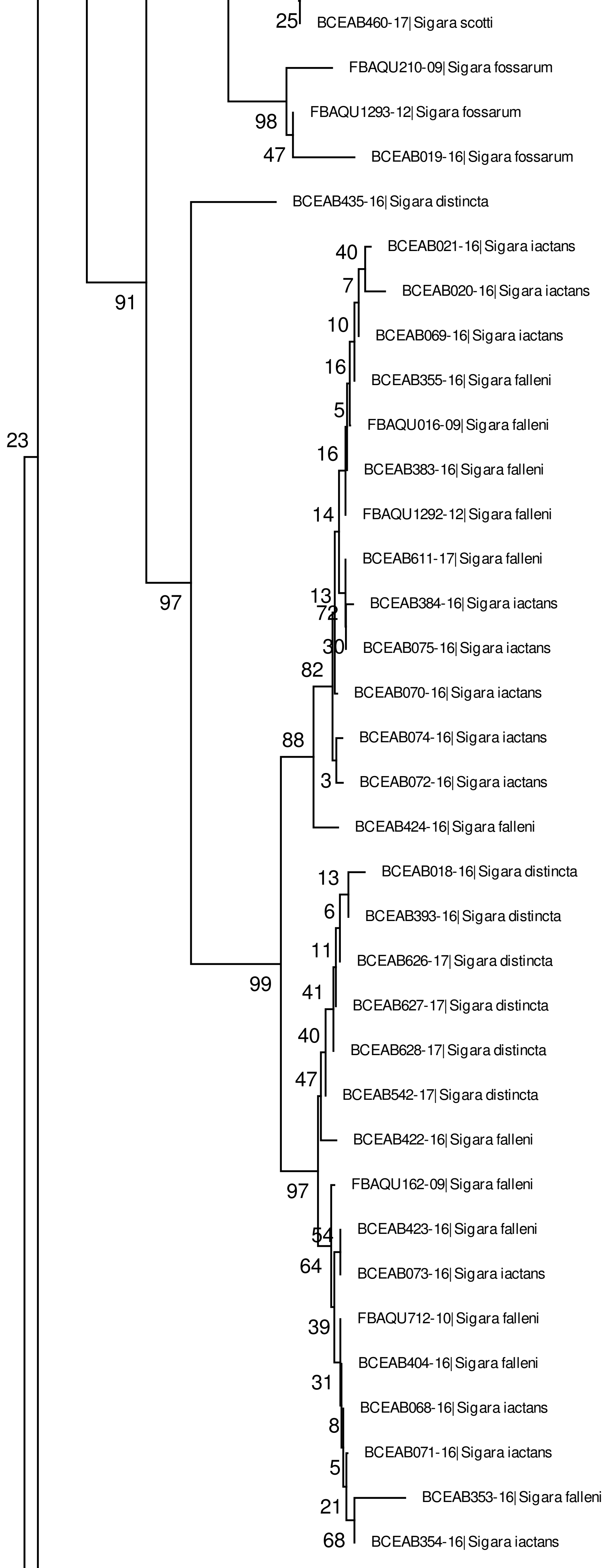
25
BCEAB460-17| Sigara scotti
FBAQU210-09| Sigara fossarum
98
FBAQU1293-12| Sigara fossarum
47
BCEAB019-16| Sigara fossarum
BCEAB435-16| Sigara distincta
40
BCEAB021-16| Sigara iactans
7
BCEAB020-16| Sigara iactans
91
10
BCEAB069-16| Sigara iactans
16
BCEAB355-16| Sigara falleni
5
FBAQU016-09| Sigara falleni
23
16
BCEAB383-16| Sigara falleni
14
FBAQU1292-12| Sigara falleni
BCEAB611-17| Sigara falleni
13
BCEAB384-16| Sigara iactans
97
72
30
BCEAB075-16| Sigara iactans
82
BCEAB070-16| Sigara iactans
88
BCEAB074-16| Sigara iactans
3
BCEAB072-16| Sigara iactans
BCEAB424-16| Sigara falleni
13
BCEAB018-16| Sigara distincta
6
BCEAB393-16| Sigara distincta
11
BCEAB626-17| Sigara distincta
99
41
BCEAB627-17| Sigara distincta
40
BCEAB628-17| Sigara distincta
47
BCEAB542-17| Sigara distincta
BCEAB422-16| Sigara falleni
97
FBAQU162-09| Sigara falleni
54
BCEAB423-16| Sigara falleni
64
BCEAB073-16| Sigara iactans
39
FBAQU712-10| Sigara falleni
BCEAB404-16| Sigara falleni
31
BCEAB068-16| Sigara iactans
8
BCEAB071-16| Sigara iactans
5
BCEAB353-16| Sigara falleni
21
68
BCEAB354-16| Sigara iactans

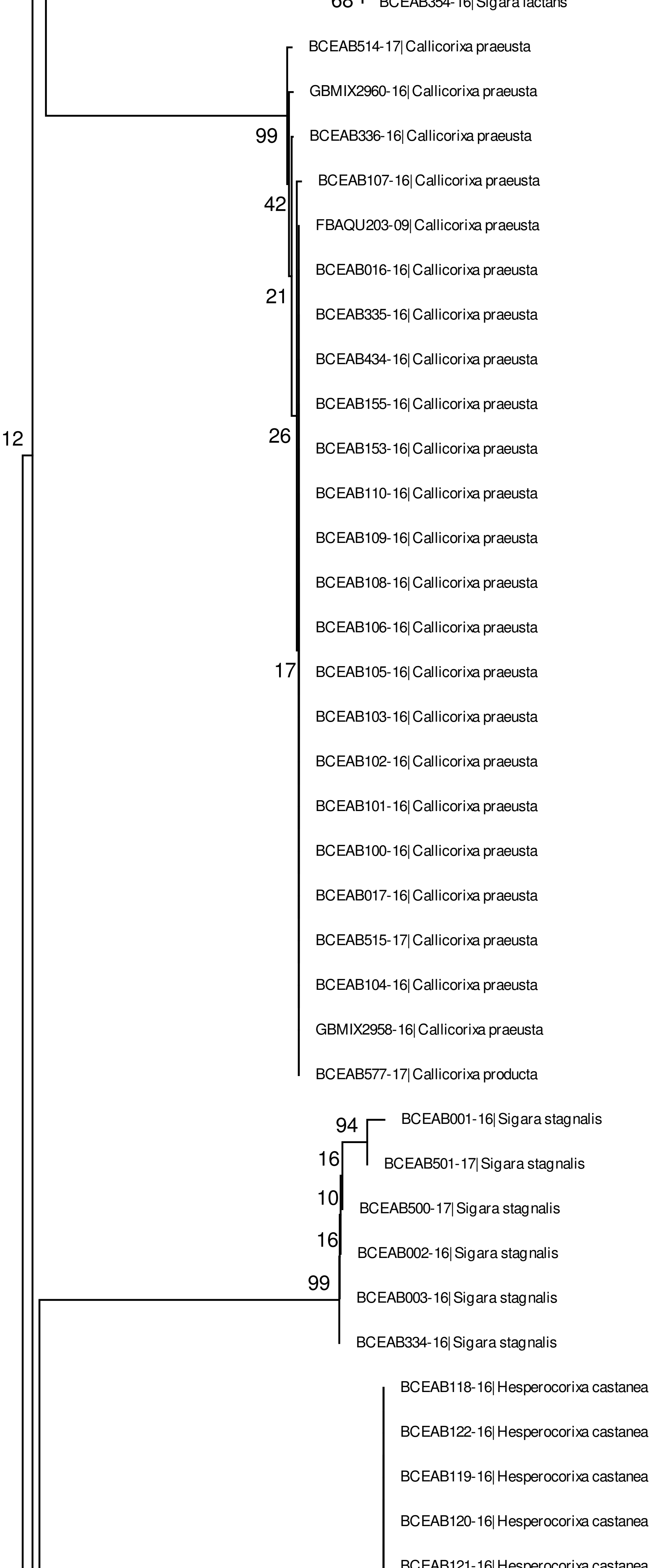
68
BCEAB354-16| Sigara lactans
BCEAB514-17| Callicorixa praeusta
GBMIX2960-16| Callicorixa praeusta
99
BCEAB336-16| Callicorixa praeusta
BCEAB107-16| Callicorixa praeusta
42
FBAQU203-09| Callicorixa praeusta
BCEAB016-16| Callicorixa praeusta
21
BCEAB335-16| Callicorixa praeusta
BCEAB434-16| Callicorixa praeusta
BCEAB155-16| Callicorixa praeusta
12
26
BCEAB153-16| Callicorixa praeusta
BCEAB110-16| Callicorixa praeusta
BCEAB109-16| Callicorixa praeusta
BCEAB108-16| Callicorixa praeusta
BCEAB106-16| Callicorixa praeusta
17
BCEAB105-16| Callicorixa praeusta
BCEAB103-16| Callicorixa praeusta
BCEAB102-16| Callicorixa praeusta
BCEAB101-16| Callicorixa praeusta
BCEAB100-16| Callicorixa praeusta
BCEAB017-16| Callicorixa praeusta
BCEAB515-17| Callicorixa praeusta
BCEAB104-16| Callicorixa praeusta
GBMIX2958-16| Callicorixa praeusta
BCEAB577-17| Callicorixa producta
94
BCEAB001-16| Sigara stagnalis
16
BCEAB501-17| Sigara stagnalis
10
BCEAB500-17| Sigara stagnalis
16
BCEAB002-16| Sigara stagnalis
99
BCEAB003-16| Sigara stagnalis
BCEAB334-16| Sigara stagnalis
BCEAB118-16| Hesperocorixa castanea
BCEAB122-16| Hesperocorixa castanea
BCEAB119-16| Hesperocorixa castanea
BCEAB120-16| Hesperocorixa castanea
BCEAB121-16| Hesperocorixa castanea

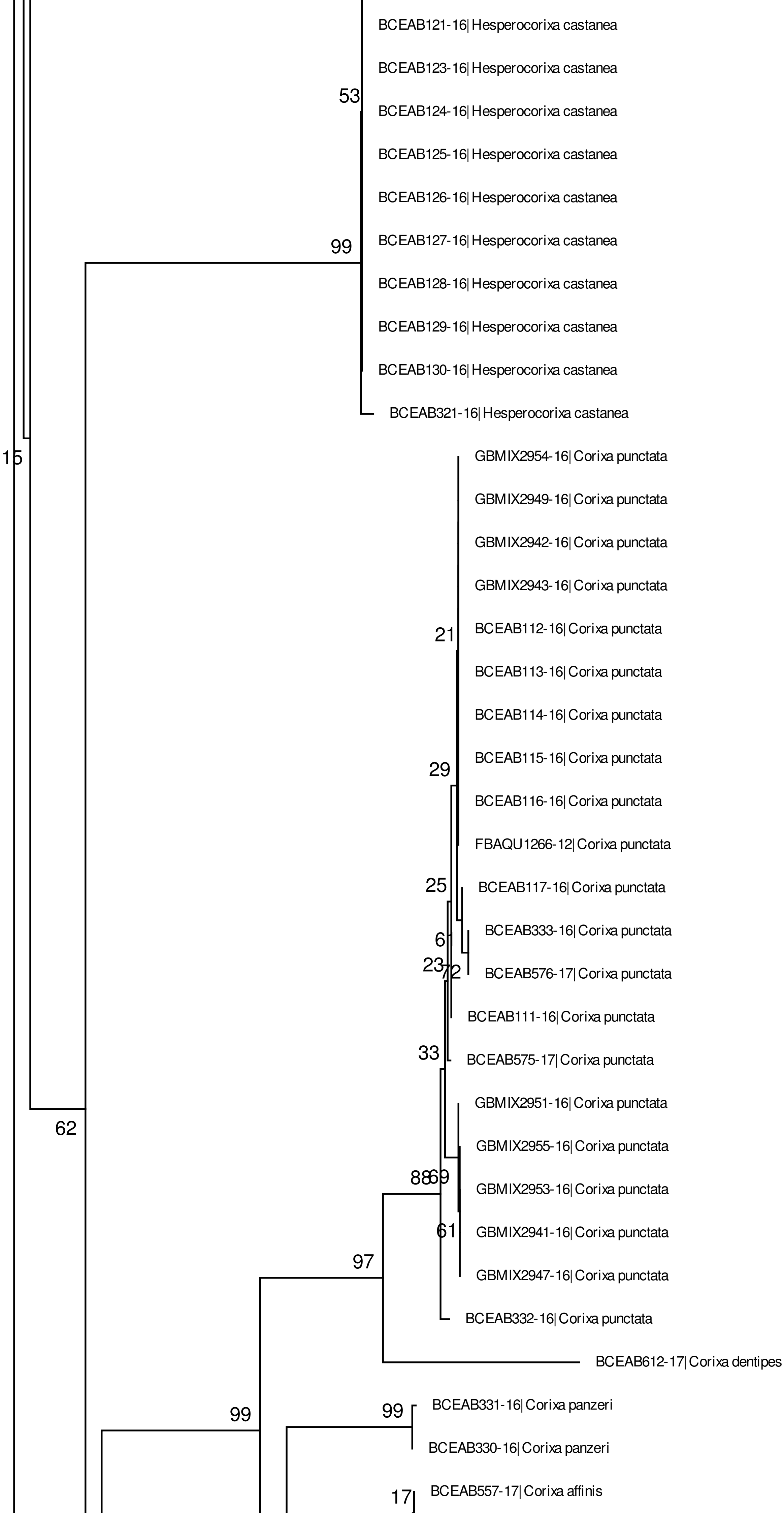

BCEAB121-16| Hesperocorixa castanea
BCEAB123-16| Hesperocorixa castanea
53
BCEAB124-16| Hesperocorixa castanea
BCEAB125-16| Hesperocorixa castanea
BCEAB126-16| Hesperocorixa castanea
99
BCEAB127-16| Hesperocorixa castanea
BCEAB128-16| Hesperocorixa castanea
BCEAB129-16| Hesperocorixa castanea
BCEAB130-16| Hesperocorixa castanea
BCEAB321-16| Hesperocorixa castanea
15
GBMIX2954-16| Corixa punctata
GBMIX2949-16| Corixa punctata
GBMIX2942-16| Corixa punctata
GBMIX2943-16| Corixa punctata
21
BCEAB112-16| Corixa punctata
BCEAB113-16| Corixa punctata
BCEAB114-16| Corixa punctata
29
BCEAB115-16| Corixa punctata
BCEAB116-16| Corixa punctata
FBAQU1266-12| Corixa punctata
25
BCEAB117-16| Corixa punctata
6
BCEAB333-16| Corixa punctata
23
72
BCEAB576-17| Corixa punctata
BCEAB111-16| Corixa punctata
33
BCEAB575-17| Corixa punctata
GBMIX2951-16| Corixa punctata
62
GBMIX2955-16| Corixa punctata
88
69
GBMIX2953-16| Corixa punctata
61
GBMIX2941-16| Corixa punctata
97
GBMIX2947-16| Corixa punctata
BCEAB332-16| Corixa punctata
BCEAB612-17| Corixa dentipes
99
BCEAB331-16| Corixa panzeri
99
BCEAB330-16| Corixa panzeri
17
BCEAB557-17| Corixa affinis

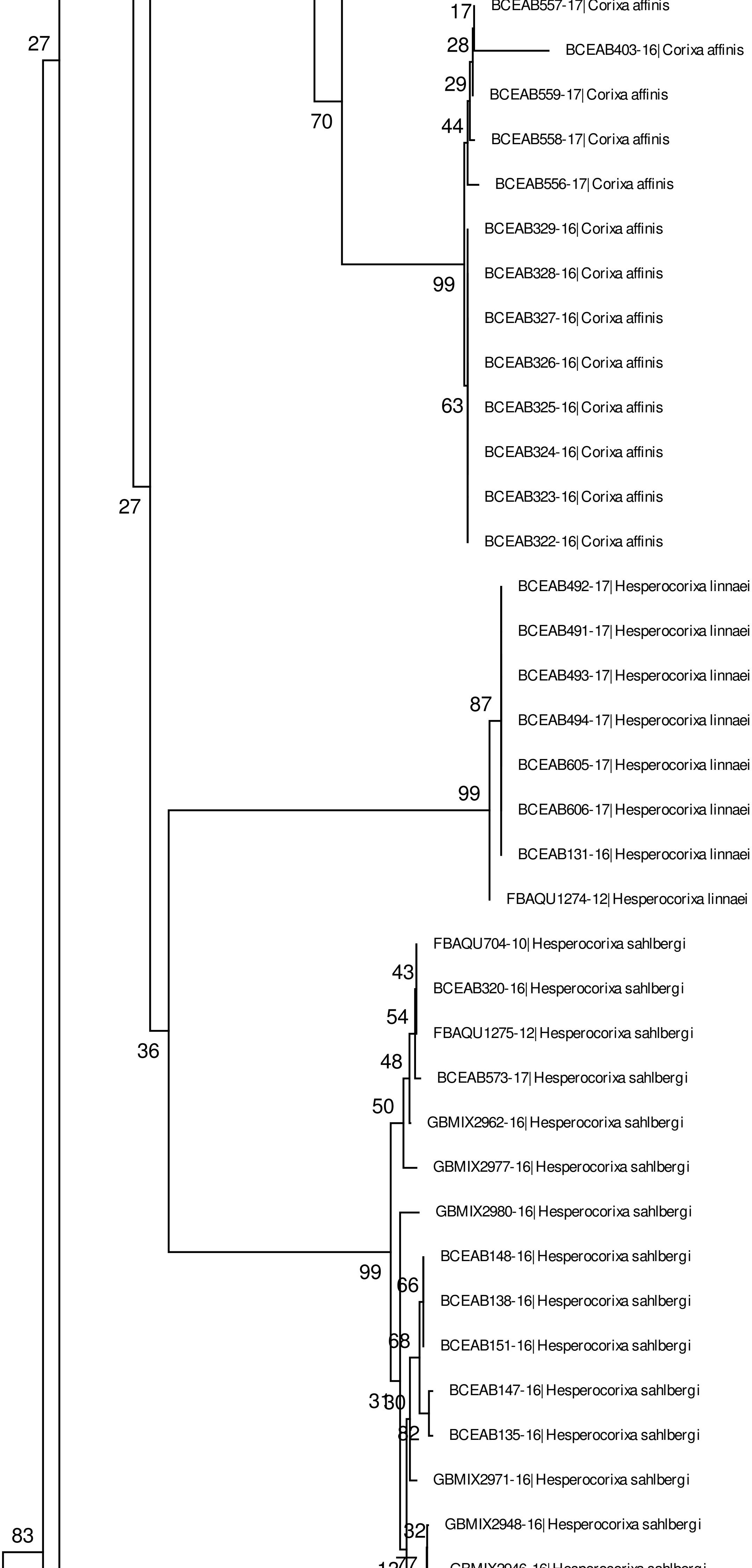
BCEAB557-17| Corixa affinis
BCEAB403-16| Corixa affinis
BCEAB559-17| Corixa affinis
BCEAB558-17| Corixa affinis
BCEAB556-17| Corixa affinis
BCEAB329-16| Corixa affinis
BCEAB328-16| Corixa affinis
BCEAB327-16| Corixa affinis
BCEAB326-16| Corixa affinis
BCEAB325-16| Corixa affinis
BCEAB324-16| Corixa affinis
BCEAB323-16| Corixa affinis
BCEAB322-16| Corixa affinis
BCEAB492-17| Hesperocorixa linnaei
BCEAB491-17| Hesperocorixa linnaei
BCEAB493-17| Hesperocorixa linnaei
BCEAB494-17| Hesperocorixa linnaei
BCEAB605-17| Hesperocorixa linnaei
BCEAB606-17| Hesperocorixa linnaei
BCEAB131-16| Hesperocorixa linnaei
FBAQU1274-12| Hesperocorixa linnaei
FBAQU704-10| Hesperocorixa sahlbergi
BCEAB320-16| Hesperocorixa sahlbergi
FBAQU1275-12| Hesperocorixa sahlbergi
BCEAB573-17| Hesperocorixa sahlbergi
GBMIX2962-16| Hesperocorixa sahlbergi
GBMIX2977-16| Hesperocorixa sahlbergi
GBMIX2980-16| Hesperocorixa sahlbergi
BCEAB148-16| Hesperocorixa sahlbergi
BCEAB138-16| Hesperocorixa sahlbergi
BCEAB151-16| Hesperocorixa sahlbergi
BCEAB147-16| Hesperocorixa sahlbergi
BCEAB135-16| Hesperocorixa sahlbergi
GBMIX2971-16| Hesperocorixa sahlbergi
GBMIX2948-16| Hesperocorixa sahlbergi
27
17
28
29
70
44
99
63
27
87
99
43
54
48
50
36
99
66
68
31
30
82
32
83

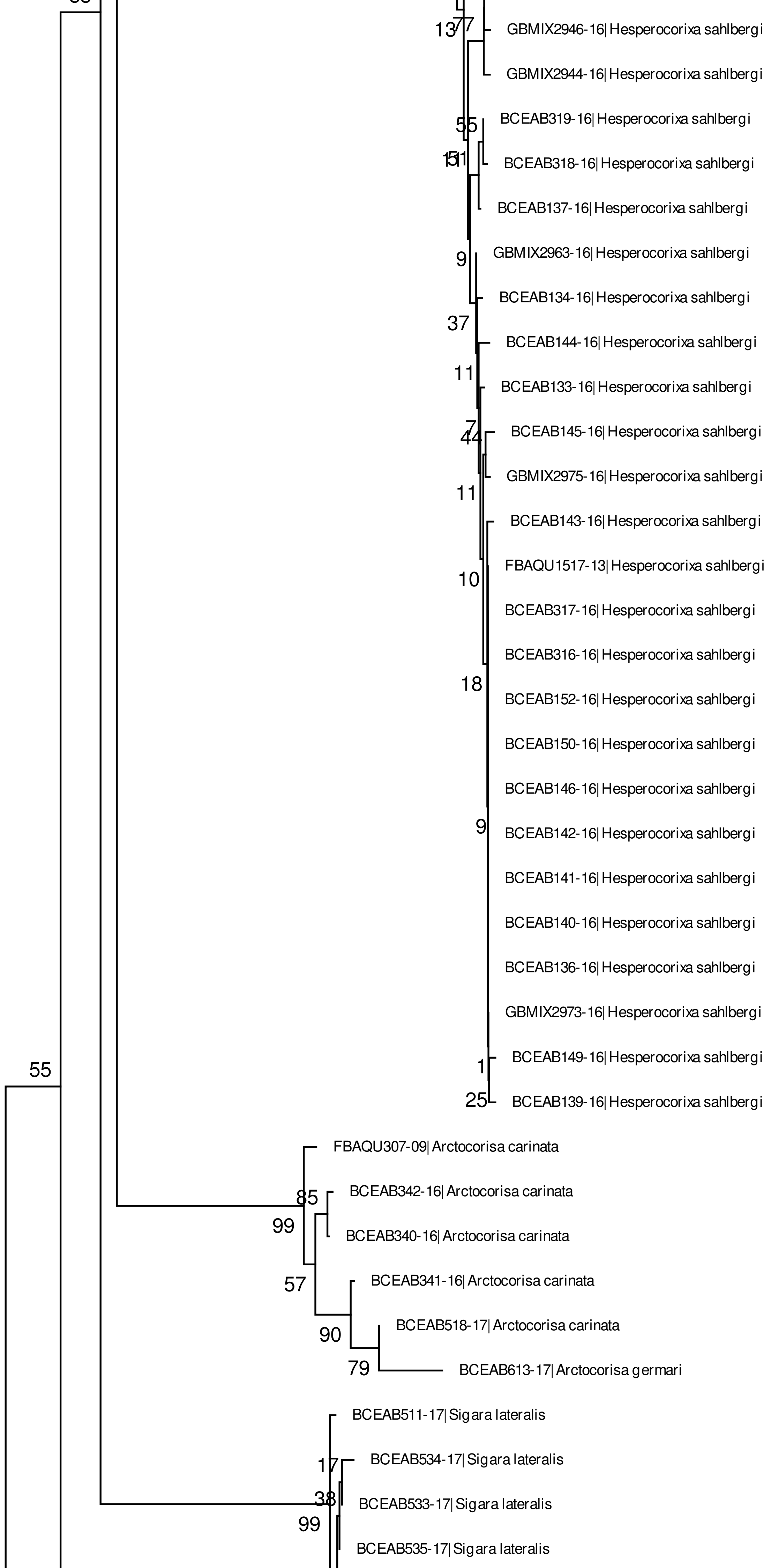

GBMIX2946-16| Hesperocorixa sahlbergi
GBMIX2944-16| Hesperocorixa sahlbergi
BCEAB319-16| Hesperocorixa sahlbergi
BCEAB318-16| Hesperocorixa sahlbergi
BCEAB137-16| Hesperocorixa sahlbergi
GBMIX2963-16| Hesperocorixa sahlbergi
BCEAB134-16| Hesperocorixa sahlbergi
BCEAB144-16| Hesperocorixa sahlbergi
BCEAB133-16| Hesperocorixa sahlbergi
BCEAB145-16| Hesperocorixa sahlbergi
GBMIX2975-16| Hesperocorixa sahlbergi
BCEAB143-16| Hesperocorixa sahlbergi
FBAQU1517-13| Hesperocorixa sahlbergi
BCEAB317-16| Hesperocorixa sahlbergi
BCEAB316-16| Hesperocorixa sahlbergi
BCEAB152-16| Hesperocorixa sahlbergi
BCEAB150-16| Hesperocorixa sahlbergi
BCEAB146-16| Hesperocorixa sahlbergi
BCEAB142-16| Hesperocorixa sahlbergi
BCEAB141-16| Hesperocorixa sahlbergi
BCEAB140-16| Hesperocorixa sahlbergi
BCEAB136-16| Hesperocorixa sahlbergi
GBMIX2973-16| Hesperocorixa sahlbergi
BCEAB149-16| Hesperocorixa sahlbergi
BCEAB139-16| Hesperocorixa sahlbergi
FBAQU307-09| Arctocorisa carinata
BCEAB342-16| Arctocorisa carinata
BCEAB340-16| Arctocorisa carinata
BCEAB341-16| Arctocorisa carinata
BCEAB518-17| Arctocorisa carinata
BCEAB613-17| Arctocorisa germari
BCEAB511-17| Sigara lateralis
BCEAB534-17| Sigara lateralis
BCEAB533-17| Sigara lateralis
BCEAB535-17| Sigara lateralis
13
77
55
9
37
11
11
10
18
9
1
25
55
85
99
57
90
79
17
38
99

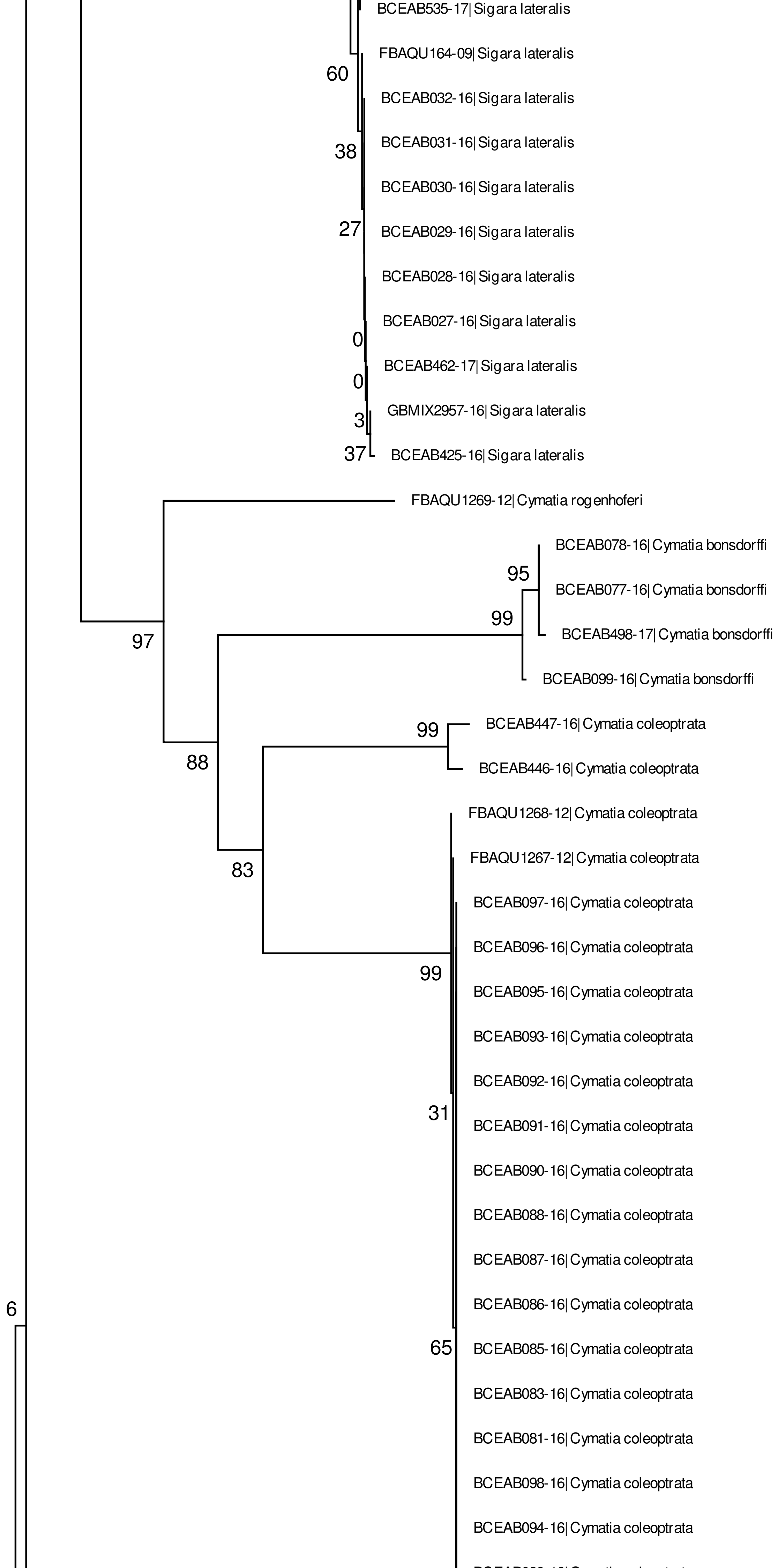
BCEAB535-17| Sigara lateralis
FBAQU164-09| Sigara lateralis
60
BCEAB032-16| Sigara lateralis
38
BCEAB031-16| Sigara lateralis
BCEAB030-16| Sigara lateralis
27
BCEAB029-16| Sigara lateralis
BCEAB028-16| Sigara lateralis
BCEAB027-16| Sigara lateralis
0
BCEAB462-17| Sigara lateralis
0
GBMIX2957-16| Sigara lateralis
3
37
BCEAB425-16| Sigara lateralis
FBAQU1269-12| Cymatia rogenhoferi
BCEAB078-16| Cymatia bonsdorffi
95
BCEAB077-16| Cymatia bonsdorffi
99
BCEAB498-17| Cymatia bonsdorffi
97
BCEAB099-16| Cymatia bonsdorffi
99
BCEAB447-16| Cymatia coleoptrata
88
BCEAB446-16| Cymatia coleoptrata
FBAQU1268-12| Cymatia coleoptrata
83
FBAQU1267-12| Cymatia coleoptrata
BCEAB097-16| Cymatia coleoptrata
BCEAB096-16| Cymatia coleoptrata
99
BCEAB095-16| Cymatia coleoptrata
BCEAB093-16| Cymatia coleoptrata
BCEAB092-16| Cymatia coleoptrata
31
BCEAB091-16| Cymatia coleoptrata
BCEAB090-16| Cymatia coleoptrata
BCEAB088-16| Cymatia coleoptrata
BCEAB087-16| Cymatia coleoptrata
6
BCEAB086-16| Cymatia coleoptrata
65
BCEAB085-16| Cymatia coleoptrata
BCEAB083-16| Cymatia coleoptrata
BCEAB081-16| Cymatia coleoptrata
BCEAB098-16| Cymatia coleoptrata
BCEAB094-16| Cymatia coleoptrata

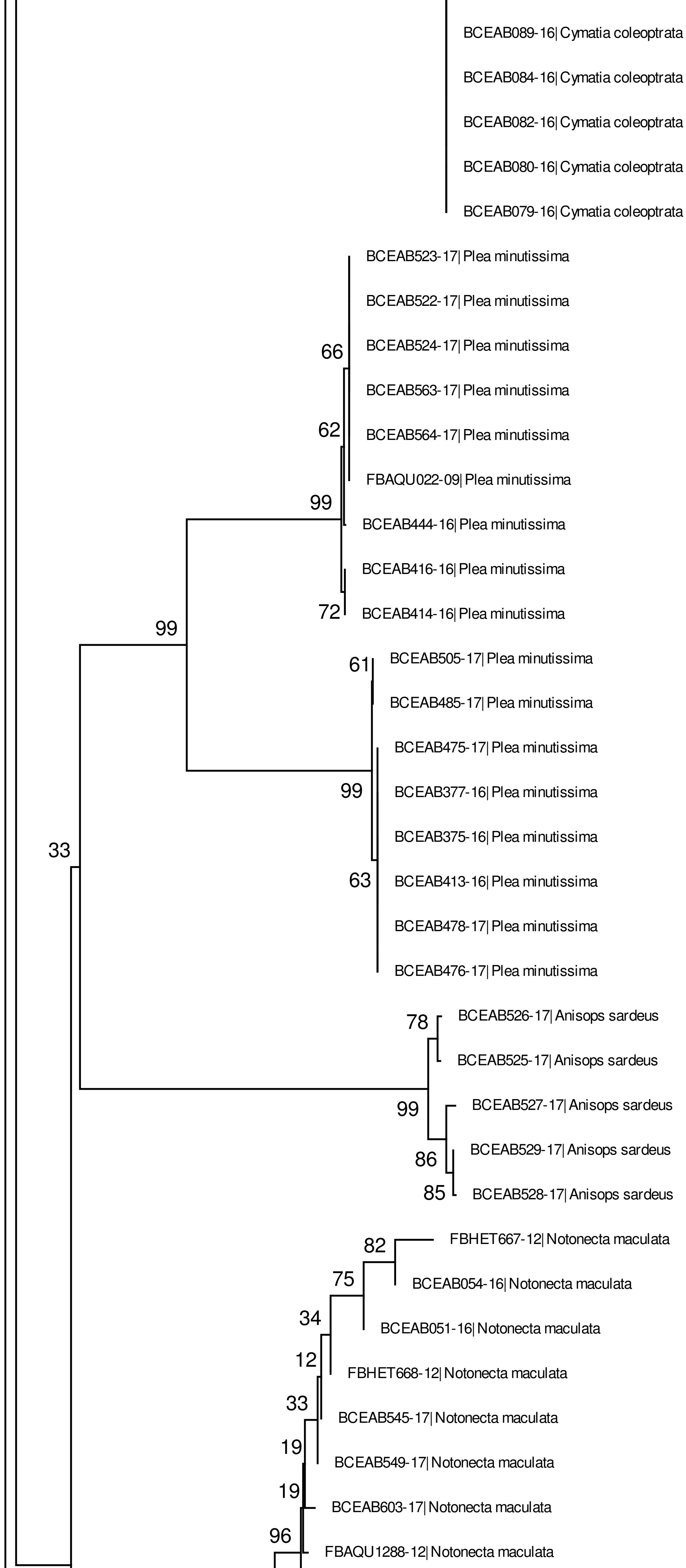
BCEAB089-16| Cymatia coleoptrata
BCEAB084-16| Cymatia coleoptrata
BCEAB082-16| Cymatia coleoptrata
BCEAB080-16| Cymatia coleoptrata
BCEAB079-16| Cymatia coleoptrata
BCEAB523-17| Plea minutissima
BCEAB522-17| Plea minutissima
66
BCEAB524-17| Plea minutissima
BCEAB563-17| Plea minutissima
62
BCEAB564-17| Plea minutissima
FBAQU022-09| Plea minutissima
99
BCEAB444-16| Plea minutissima
BCEAB416-16| Plea minutissima
99
72
BCEAB414-16| Plea minutissima
61
BCEAB505-17| Plea minutissima
BCEAB485-17| Plea minutissima
BCEAB475-17| Plea minutissima
99
BCEAB377-16| Plea minutissima
33
BCEAB375-16| Plea minutissima
63
BCEAB413-16| Plea minutissima
BCEAB478-17| Plea minutissima
BCEAB476-17| Plea minutissima
78
BCEAB526-17| Anisops sardeus
BCEAB525-17| Anisops sardeus
99
BCEAB527-17| Anisops sardeus
86
BCEAB529-17| Anisops sardeus
85
BCEAB528-17| Anisops sardeus
82
FBHET667-12| Notonecta maculata
75
BCEAB054-16| Notonecta maculata
34
BCEAB051-16| Notonecta maculata
12
FBHET668-12| Notonecta maculata
33
BCEAB545-17| Notonecta maculata
19
BCEAB549-17| Notonecta maculata
19
BCEAB603-17| Notonecta maculata
96
FBAQU1288-12| Notonecta maculata

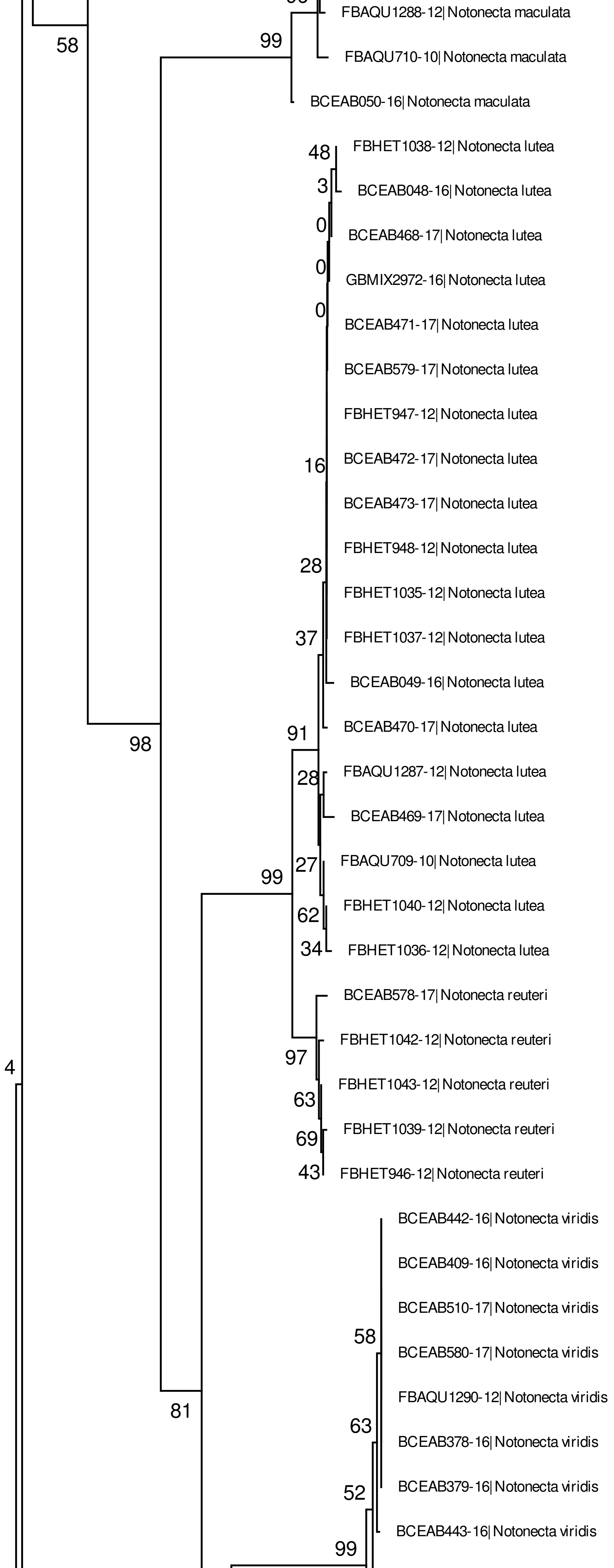
FBAQU1288-12|Notonecta maculata
58
99
FBAQU710-10|Notonecta maculata
BCEAB050-16|Notonecta maculata
48
FBHET1038-12|Notonecta lutea
3
BCEAB048-16|Notonecta lutea
0
BCEAB468-17|Notonecta lutea
0
GBMIX2972-16|Notonecta lutea
0
BCEAB471-17|Notonecta lutea
BCEAB579-17|Notonecta lutea
FBHET947-12|Notonecta lutea
16
BCEAB472-17|Notonecta lutea
BCEAB473-17|Notonecta lutea
FBHET948-12|Notonecta lutea
28
FBHET1035-12|Notonecta lutea
37
FBHET1037-12|Notonecta lutea
BCEAB049-16|Notonecta lutea
98
91
BCEAB470-17|Notonecta lutea
28
FBAQU1287-12|Notonecta lutea
BCEAB469-17|Notonecta lutea
99
27
FBAQU709-10|Notonecta lutea
62
FBHET1040-12|Notonecta lutea
34
FBHET1036-12|Notonecta lutea
BCEAB578-17|Notonecta reuteri
FBHET1042-12|Notonecta reuteri
4
97
63
FBHET1043-12|Notonecta reuteri
69
FBHET1039-12|Notonecta reuteri
43
FBHET946-12|Notonecta reuteri
BCEAB442-16|Notonecta viridis
BCEAB409-16|Notonecta viridis
BCEAB510-17|Notonecta viridis
58
BCEAB580-17|Notonecta viridis
FBAQU1290-12|Notonecta viridis
81
63
BCEAB378-16|Notonecta viridis
52
BCEAB379-16|Notonecta viridis
BCEAB443-16|Notonecta viridis
99

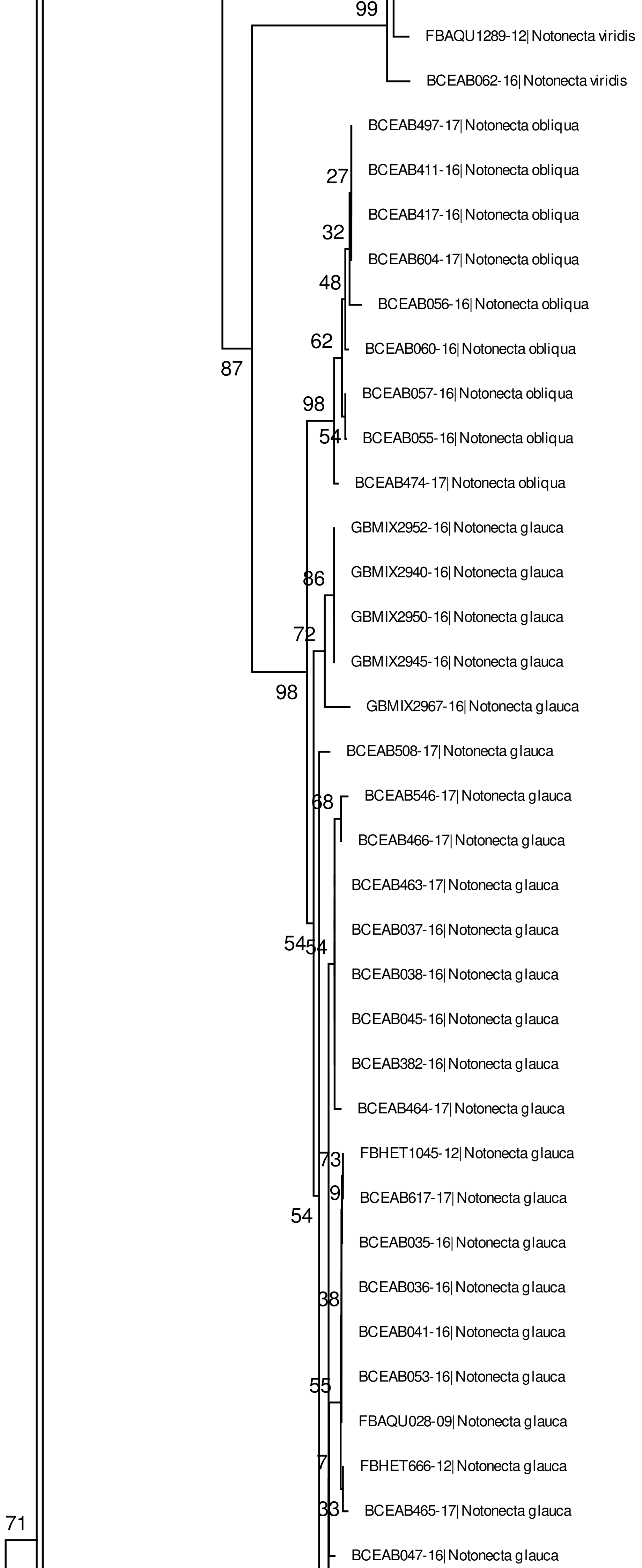
99
FBAQU1289-12| Notonecta viridis
BCEAB062-16| Notonecta viridis
BCEAB497-17| Notonecta obliqua
27
BCEAB411-16| Notonecta obliqua
32
BCEAB417-16| Notonecta obliqua
48
BCEAB604-17| Notonecta obliqua
BCEAB056-16| Notonecta obliqua
62
BCEAB060-16| Notonecta obliqua
87
98
BCEAB057-16| Notonecta obliqua
54
BCEAB055-16| Notonecta obliqua
BCEAB474-17| Notonecta obliqua
GBMIX2952-16| Notonecta glauca
86
GBMIX2940-16| Notonecta glauca
GBMIX2950-16| Notonecta glauca
72
GBMIX2945-16| Notonecta glauca
98
GBMIX2967-16| Notonecta glauca
BCEAB508-17| Notonecta glauca
68
BCEAB546-17| Notonecta glauca
BCEAB466-17| Notonecta glauca
BCEAB463-17| Notonecta glauca
BCEAB037-16| Notonecta glauca
54
54
BCEAB038-16| Notonecta glauca
BCEAB045-16| Notonecta glauca
BCEAB382-16| Notonecta glauca
BCEAB464-17| Notonecta glauca
73
FBHET1045-12| Notonecta glauca
9
BCEAB617-17| Notonecta glauca
54
BCEAB035-16| Notonecta glauca
38
BCEAB036-16| Notonecta glauca
BCEAB041-16| Notonecta glauca
55
BCEAB053-16| Notonecta glauca
FBAQU028-09| Notonecta glauca
7
FBHET666-12| Notonecta glauca
71
33
BCEAB465-17| Notonecta glauca
BCEAB047-16| Notonecta glauca

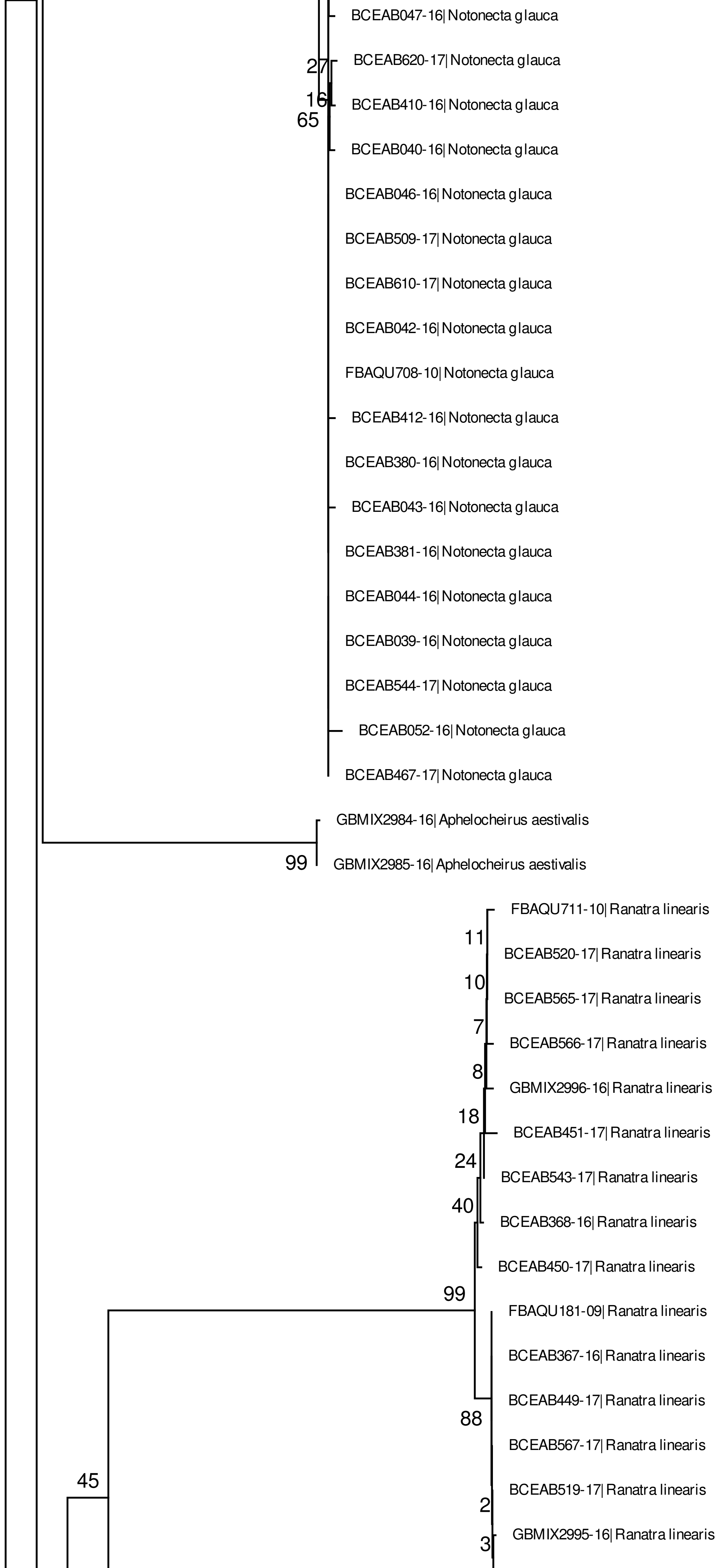
BCEAB047-16| Notonecta glauca
27
BCEAB620-17| Notonecta glauca
16
BCEAB410-16| Notonecta glauca
65
BCEAB040-16| Notonecta glauca
BCEAB046-16| Notonecta glauca
BCEAB509-17| Notonecta glauca
BCEAB610-17| Notonecta glauca
BCEAB042-16| Notonecta glauca
FBAQU708-10| Notonecta glauca
BCEAB412-16| Notonecta glauca
BCEAB380-16| Notonecta glauca
BCEAB043-16| Notonecta glauca
BCEAB381-16| Notonecta glauca
BCEAB044-16| Notonecta glauca
BCEAB039-16| Notonecta glauca
BCEAB544-17| Notonecta glauca
BCEAB052-16| Notonecta glauca
BCEAB467-17| Notonecta glauca
GBMIX2984-16| Aphelocheirus aestivalis
99
GBMIX2985-16| Aphelocheirus aestivalis
FBAQU711-10| Ranatra linearis
11
BCEAB520-17| Ranatra linearis
10
BCEAB565-17| Ranatra linearis
7
BCEAB566-17| Ranatra linearis
8
GBMIX2996-16| Ranatra linearis
18
BCEAB451-17| Ranatra linearis
24
BCEAB543-17| Ranatra linearis
40
BCEAB368-16| Ranatra linearis
BCEAB450-17| Ranatra linearis
99
FBAQU181-09| Ranatra linearis
BCEAB367-16| Ranatra linearis
BCEAB449-17| Ranatra linearis
88
BCEAB567-17| Ranatra linearis
45
BCEAB519-17| Ranatra linearis
2
GBMIX2995-16| Ranatra linearis
3

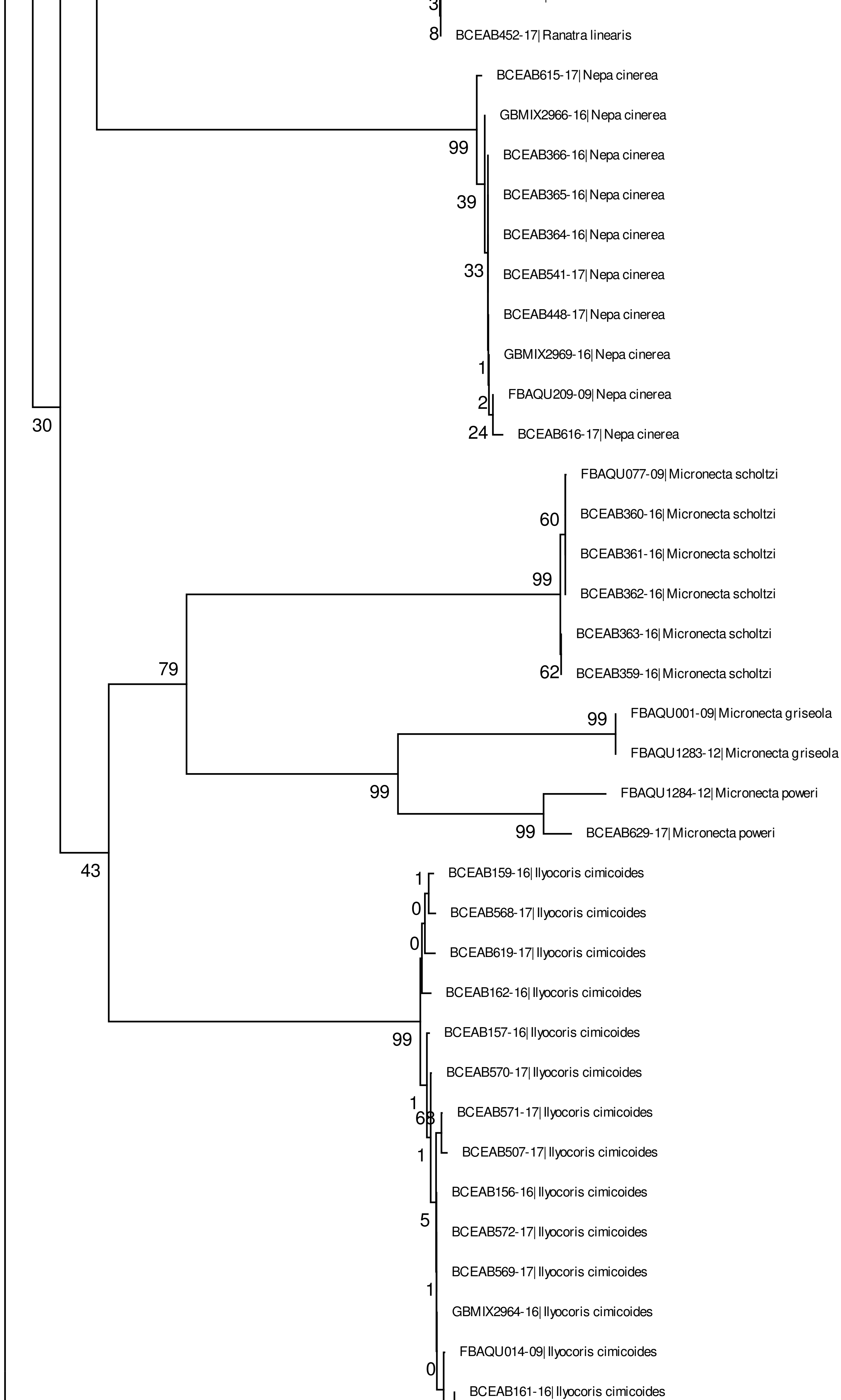
BCEAB452-17| Ranatra linearis
BCEAB615-17| Nepa cinerea
GBMIX2966-16| Nepa cinerea
BCEAB366-16| Nepa cinerea
BCEAB365-16| Nepa cinerea
BCEAB364-16| Nepa cinerea
BCEAB541-17| Nepa cinerea
BCEAB448-17| Nepa cinerea
GBMIX2969-16| Nepa cinerea
FBAQU209-09| Nepa cinerea
BCEAB616-17| Nepa cinerea
FBAQU077-09| Micronecta scholtzi
BCEAB360-16| Micronecta scholtzi
BCEAB361-16| Micronecta scholtzi
BCEAB362-16| Micronecta scholtzi
BCEAB363-16| Micronecta scholtzi
BCEAB359-16| Micronecta scholtzi
FBAQU001-09| Micronecta griseola
FBAQU1283-12| Micronecta griseola
FBAQU1284-12| Micronecta poweri
BCEAB629-17| Micronecta poweri
BCEAB159-16| Ilyocoris cimicoides
BCEAB568-17| Ilyocoris cimicoides
BCEAB619-17| Ilyocoris cimicoides
BCEAB162-16| Ilyocoris cimicoides
BCEAB157-16| Ilyocoris cimicoides
BCEAB570-17| Ilyocoris cimicoides
BCEAB571-17| Ilyocoris cimicoides
BCEAB507-17| Ilyocoris cimicoides
BCEAB156-16| Ilyocoris cimicoides
BCEAB572-17| Ilyocoris cimicoides
BCEAB569-17| Ilyocoris cimicoides
GBMIX2964-16| Ilyocoris cimicoides
FBAQU014-09| Ilyocoris cimicoides
BCEAB161-16| Ilyocoris cimicoides

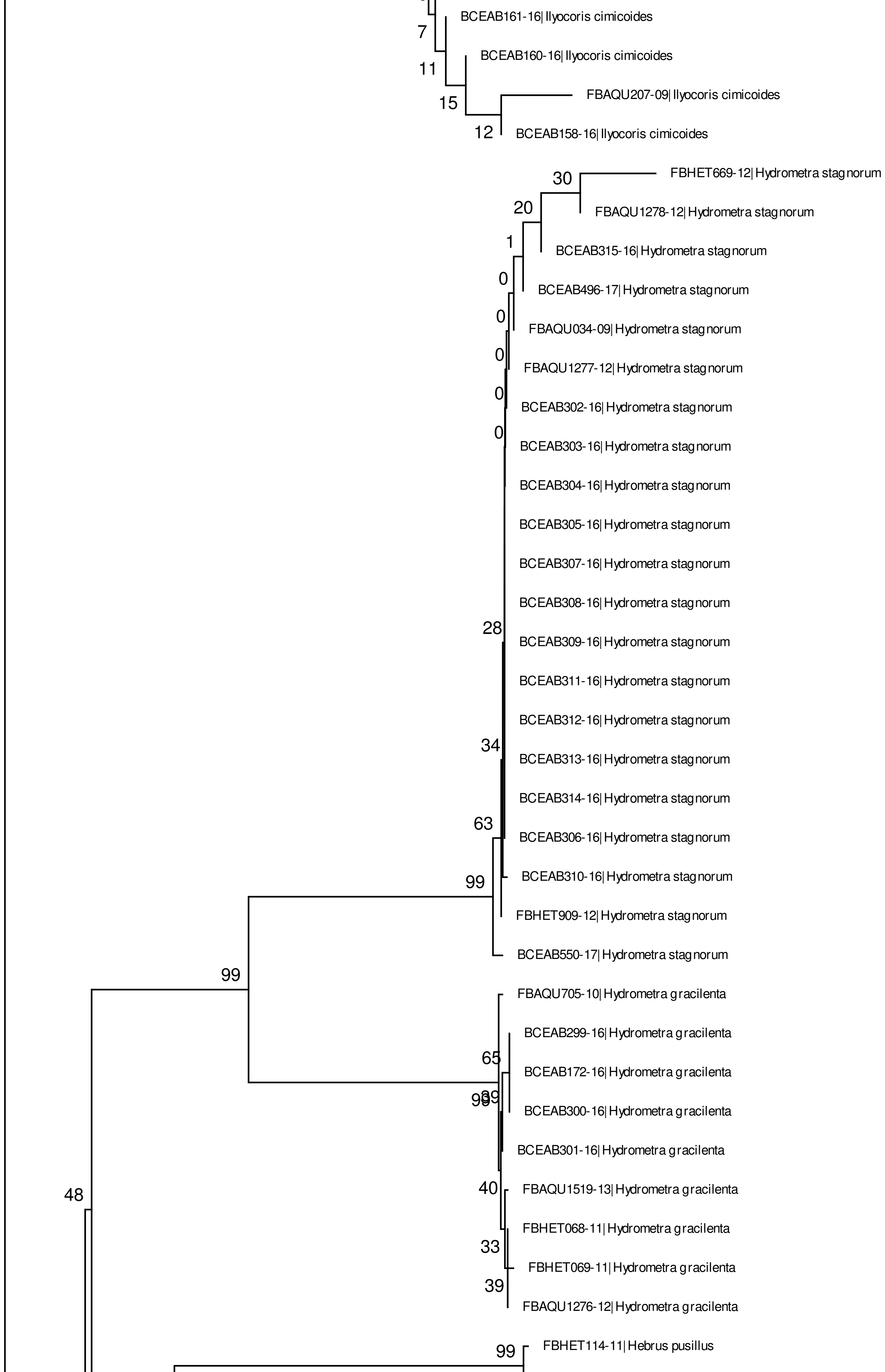

BCEAB161-16| Ilyocoris cimicoides
7
BCEAB160-16| Ilyocoris cimicoides
11
FBAQU207-09| Ilyocoris cimicoides
15
BCEAB158-16| Ilyocoris cimicoides
12
30
FBHET669-12| Hydrometra stagnorum
20
FBAQU1278-12| Hydrometra stagnorum
1
BCEAB315-16| Hydrometra stagnorum
0
BCEAB496-17| Hydrometra stagnorum
0
FBAQU034-09| Hydrometra stagnorum
0
FBAQU1277-12| Hydrometra stagnorum
0
BCEAB302-16| Hydrometra stagnorum
0
BCEAB303-16| Hydrometra stagnorum
BCEAB304-16| Hydrometra stagnorum
BCEAB305-16| Hydrometra stagnorum
BCEAB307-16| Hydrometra stagnorum
BCEAB308-16| Hydrometra stagnorum
28
BCEAB309-16| Hydrometra stagnorum
BCEAB311-16| Hydrometra stagnorum
BCEAB312-16| Hydrometra stagnorum
34
BCEAB313-16| Hydrometra stagnorum
BCEAB314-16| Hydrometra stagnorum
63
BCEAB306-16| Hydrometra stagnorum
99
BCEAB310-16| Hydrometra stagnorum
FBHET909-12| Hydrometra stagnorum
BCEAB550-17| Hydrometra stagnorum
99
FBAQU705-10| Hydrometra gracilenta
BCEAB299-16| Hydrometra gracilenta
65
BCEAB172-16| Hydrometra gracilenta
99
39
BCEAB300-16| Hydrometra gracilenta
BCEAB301-16| Hydrometra gracilenta
48
40
FBAQU1519-13| Hydrometra gracilenta
FBHET068-11| Hydrometra gracilenta
33
FBHET069-11| Hydrometra gracilenta
39
FBAQU1276-12| Hydrometra gracilenta
99
FBHET114-11| Hebrus pusillus

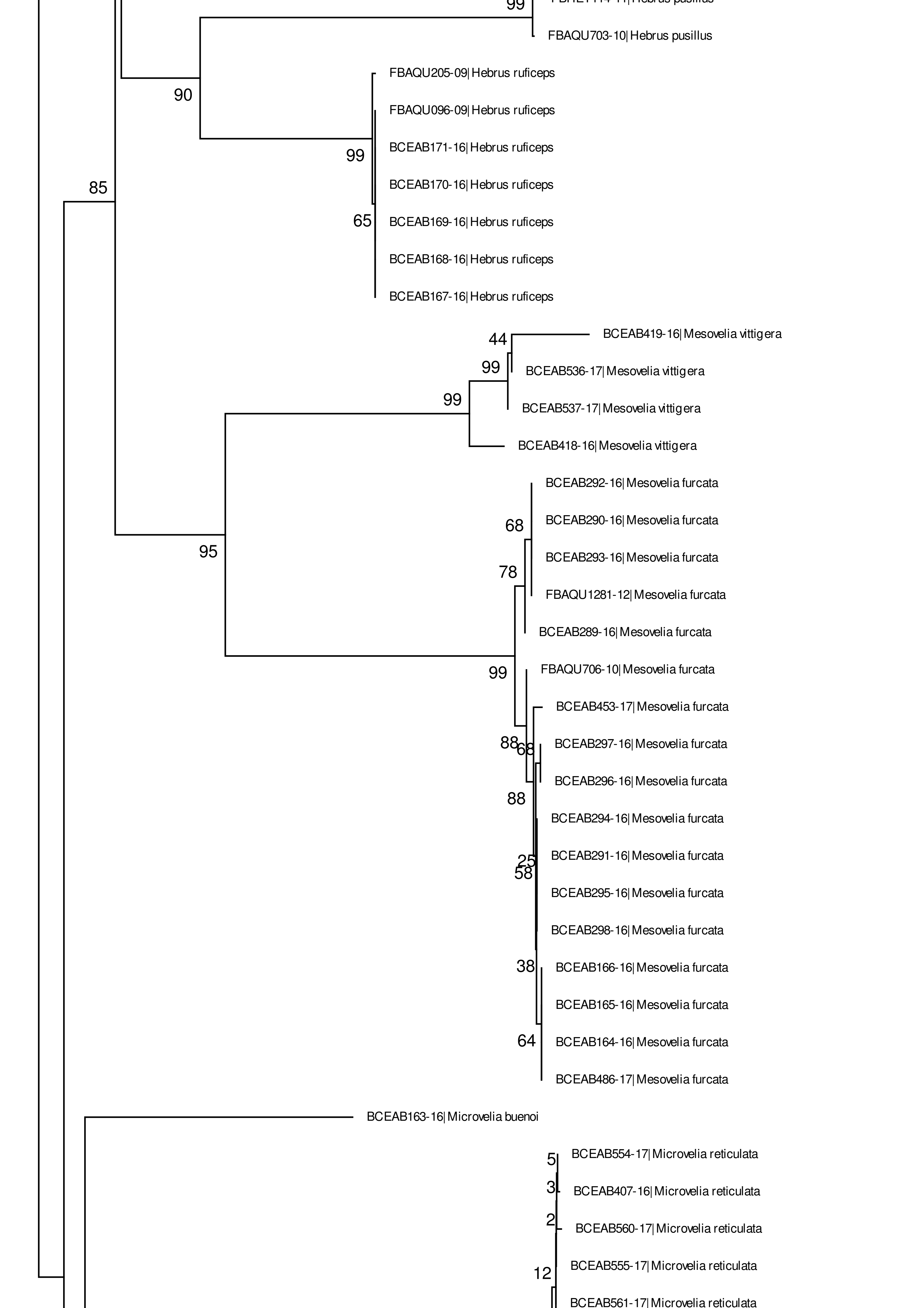
99
FBAQU703-10|Hebrus pusillus
90
FBAQU205-09|Hebrus ruficeps
FBAQU096-09|Hebrus ruficeps
99
BCEAB171-16|Hebrus ruficeps
85
BCEAB170-16|Hebrus ruficeps
65
BCEAB169-16|Hebrus ruficeps
BCEAB168-16|Hebrus ruficeps
BCEAB167-16|Hebrus ruficeps
44
BCEAB419-16|Mesovelia vittigera
99
BCEAB536-17|Mesovelia vittigera
99
BCEAB537-17|Mesovelia vittigera
BCEAB418-16|Mesovelia vittigera
BCEAB292-16|Mesovelia furcata
68
BCEAB290-16|Mesovelia furcata
95
BCEAB293-16|Mesovelia furcata
78
FBAQU1281-12|Mesovelia furcata
BCEAB289-16|Mesovelia furcata
99
FBAQU706-10|Mesovelia furcata
BCEAB453-17|Mesovelia furcata
88
68
BCEAB297-16|Mesovelia furcata
BCEAB296-16|Mesovelia furcata
88
BCEAB294-16|Mesovelia furcata
25
58
BCEAB291-16|Mesovelia furcata
BCEAB295-16|Mesovelia furcata
BCEAB298-16|Mesovelia furcata
38
BCEAB166-16|Mesovelia furcata
BCEAB165-16|Mesovelia furcata
64
BCEAB164-16|Mesovelia furcata
BCEAB486-17|Mesovelia furcata
BCEAB163-16|Microvelia buenoi
5
BCEAB554-17|Microvelia reticulata
3
BCEAB407-16|Microvelia reticulata
2
BCEAB560-17|Microvelia reticulata
12
BCEAB555-17|Microvelia reticulata
BCEAB561-17|Microvelia reticulata

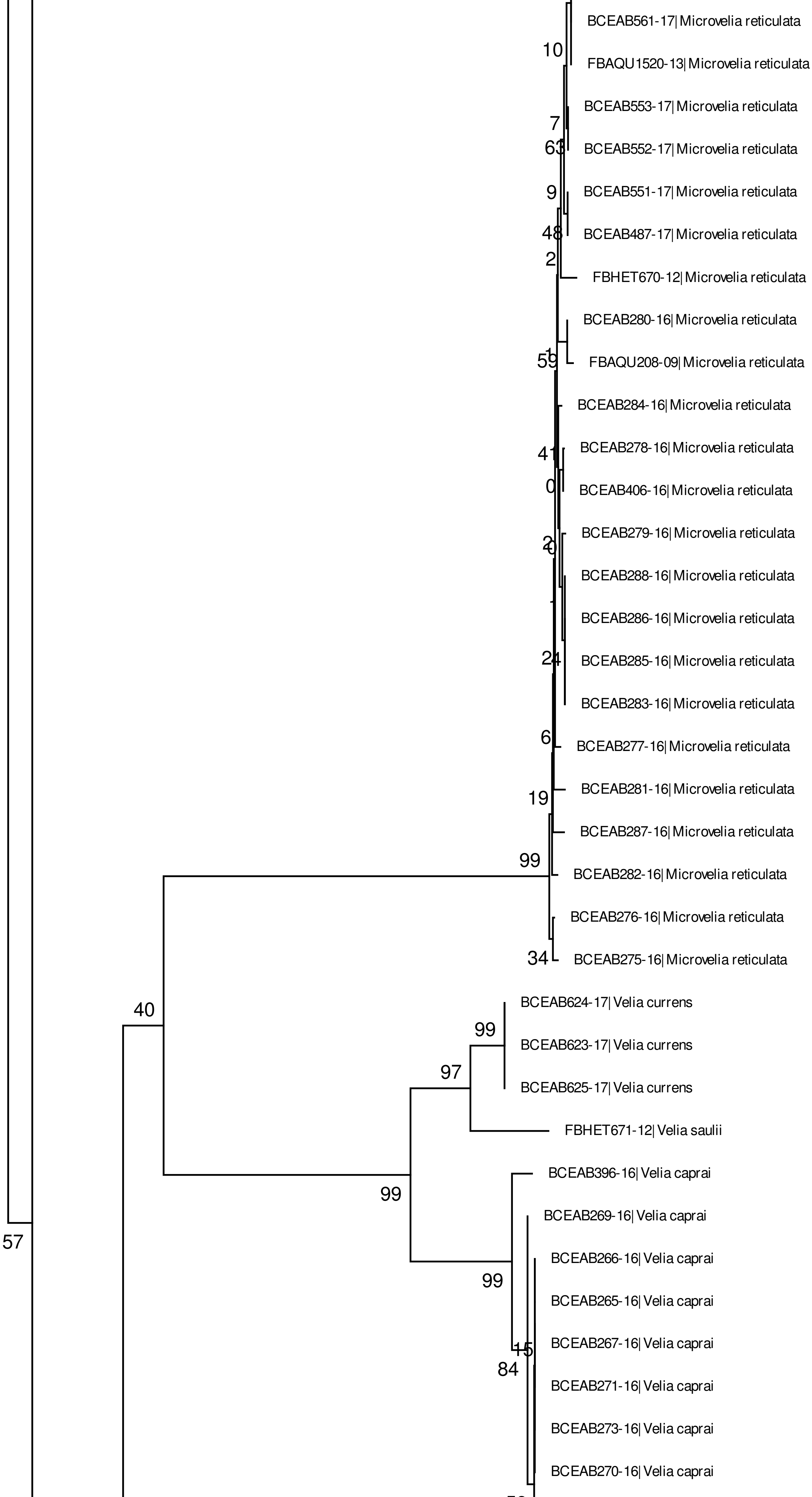
BCEAB561-17| Microvelia reticulata
FBAQU1520-13| Microvelia reticulata
BCEAB553-17| Microvelia reticulata
BCEAB552-17| Microvelia reticulata
BCEAB551-17| Microvelia reticulata
BCEAB487-17| Microvelia reticulata
FBHET670-12| Microvelia reticulata
BCEAB280-16| Microvelia reticulata
FBAQU208-09| Microvelia reticulata
BCEAB284-16| Microvelia reticulata
BCEAB278-16| Microvelia reticulata
BCEAB406-16| Microvelia reticulata
BCEAB279-16| Microvelia reticulata
BCEAB288-16| Microvelia reticulata
BCEAB286-16| Microvelia reticulata
BCEAB285-16| Microvelia reticulata
BCEAB283-16| Microvelia reticulata
BCEAB277-16| Microvelia reticulata
BCEAB281-16| Microvelia reticulata
BCEAB287-16| Microvelia reticulata
BCEAB282-16| Microvelia reticulata
BCEAB276-16| Microvelia reticulata
BCEAB275-16| Microvelia reticulata
BCEAB624-17| Velia currens
BCEAB623-17| Velia currens
BCEAB625-17| Velia currens
FBHET671-12| Velia saulii
BCEAB396-16| Velia caprai
BCEAB269-16| Velia caprai
BCEAB266-16| Velia caprai
BCEAB265-16| Velia caprai
BCEAB267-16| Velia caprai
BCEAB271-16| Velia caprai
BCEAB273-16| Velia caprai
BCEAB270-16| Velia caprai
10
7
9
2
19
99
34
40
99
97
99
57
99
84

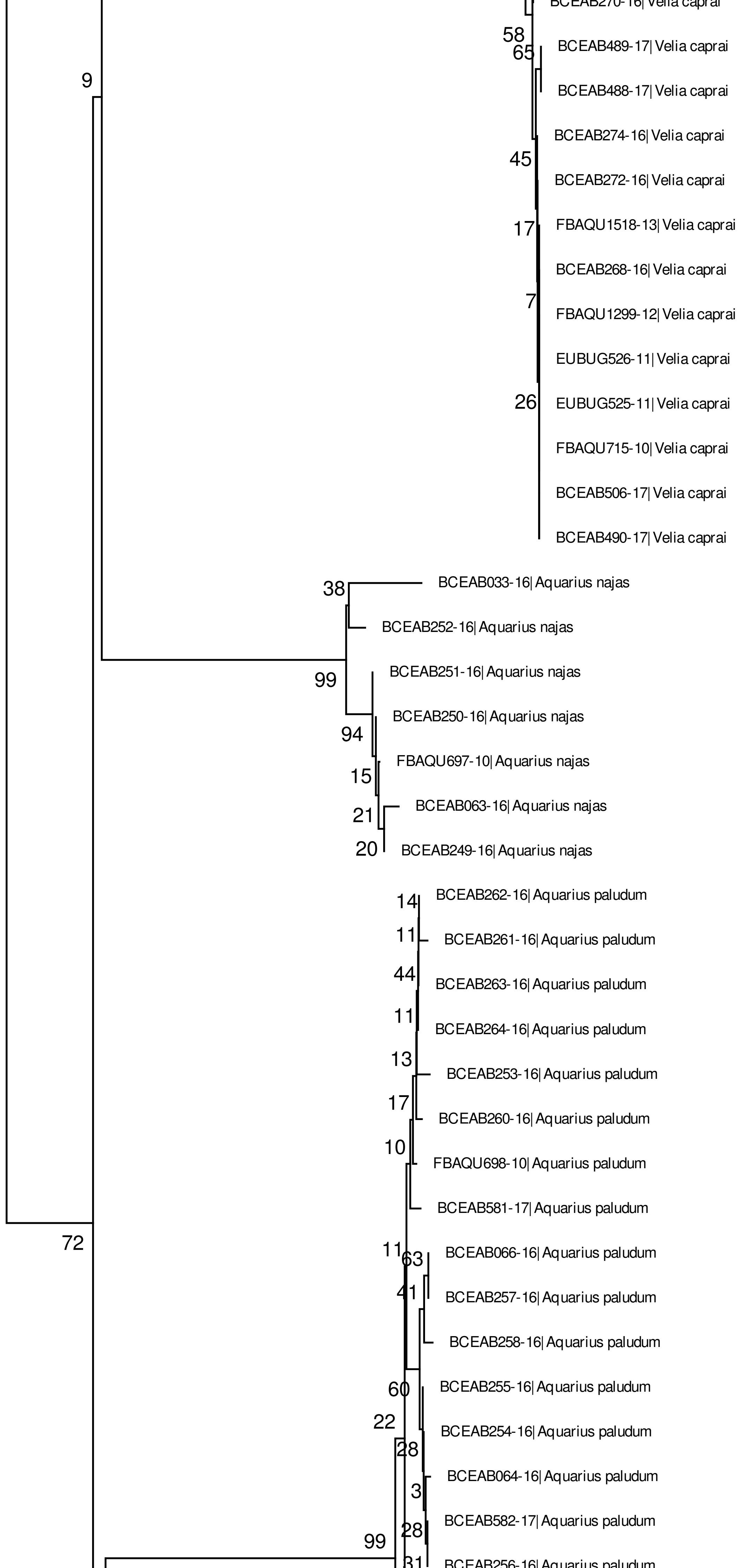
BCEAB270-16| Velia caprai
BCEAB489-17| Velia caprai
BCEAB488-17| Velia caprai
BCEAB274-16| Velia caprai
BCEAB272-16| Velia caprai
FBAQU1518-13| Velia caprai
BCEAB268-16| Velia caprai
FBAQU1299-12| Velia caprai
EUBUG526-11| Velia caprai
EUBUG525-11| Velia caprai
FBAQU715-10| Velia caprai
BCEAB506-17| Velia caprai
BCEAB490-17| Velia caprai
BCEAB033-16| Aquarius najas
BCEAB252-16| Aquarius najas
BCEAB251-16| Aquarius najas
BCEAB250-16| Aquarius najas
FBAQU697-10| Aquarius najas
BCEAB063-16| Aquarius najas
BCEAB249-16| Aquarius najas
BCEAB262-16| Aquarius paludum
BCEAB261-16| Aquarius paludum
BCEAB263-16| Aquarius paludum
BCEAB264-16| Aquarius paludum
BCEAB253-16| Aquarius paludum
BCEAB260-16| Aquarius paludum
FBAQU698-10| Aquarius paludum
BCEAB581-17| Aquarius paludum
BCEAB066-16| Aquarius paludum
BCEAB257-16| Aquarius paludum
BCEAB258-16| Aquarius paludum
BCEAB255-16| Aquarius paludum
BCEAB254-16| Aquarius paludum
BCEAB064-16| Aquarius paludum
BCEAB582-17| Aquarius paludum
BCEAB256-16| Aquarius paludum

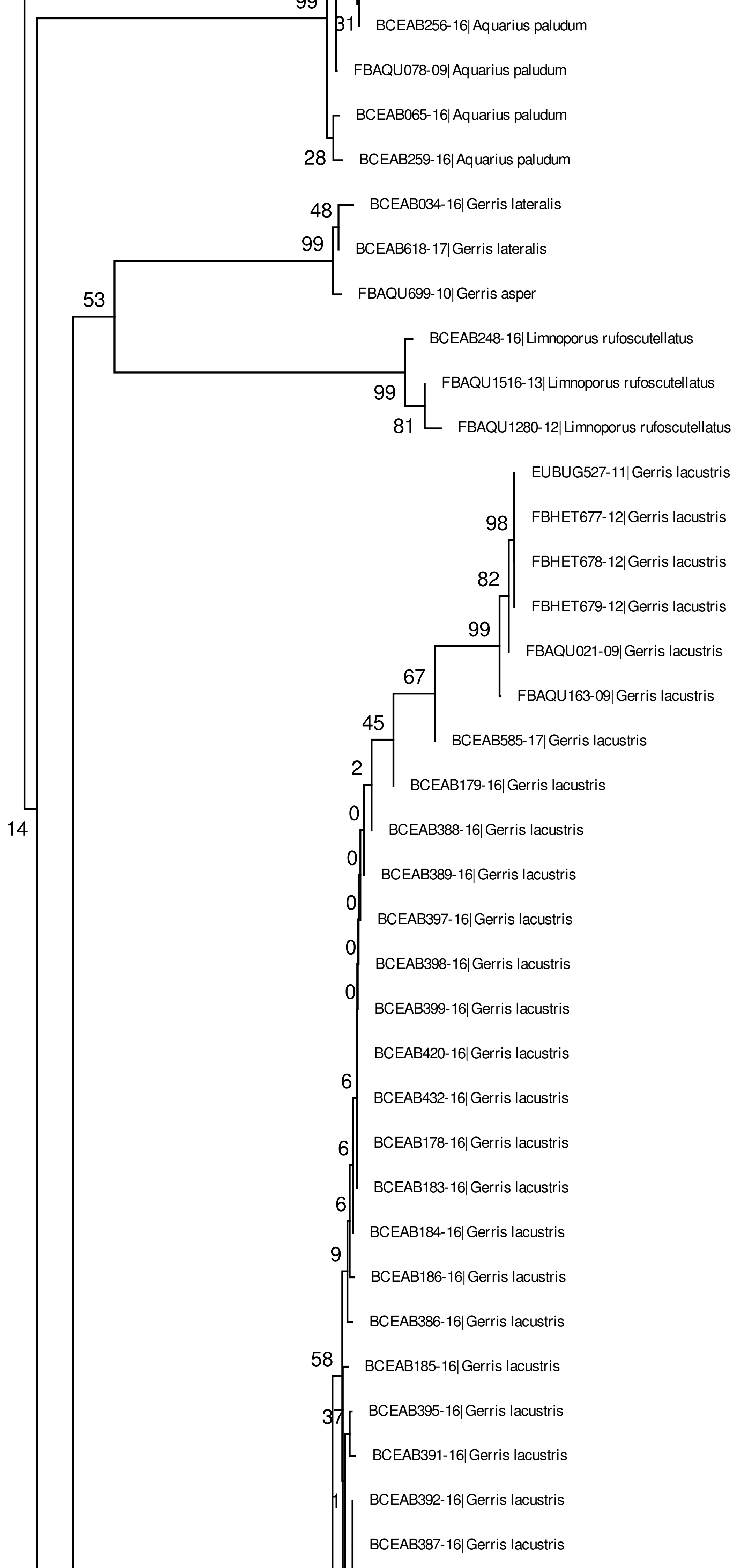
BCEAB256-16| Aquarius paludum
FBAQU078-09| Aquarius paludum
BCEAB065-16| Aquarius paludum
BCEAB259-16| Aquarius paludum
BCEAB034-16| Gerris lateralis
BCEAB618-17| Gerris lateralis
FBAQU699-10| Gerris asper
BCEAB248-16| Limnoporus rufoscutellatus
FBAQU1516-13| Limnoporus rufoscutellatus
FBAQU1280-12| Limnoporus rufoscutellatus
EUBUG527-11| Gerris lacustris
FBHET677-12| Gerris lacustris
FBHET678-12| Gerris lacustris
FBHET679-12| Gerris lacustris
FBAQU021-09| Gerris lacustris
FBAQU163-09| Gerris lacustris
BCEAB585-17| Gerris lacustris
BCEAB179-16| Gerris lacustris
BCEAB388-16| Gerris lacustris
BCEAB389-16| Gerris lacustris
BCEAB397-16| Gerris lacustris
BCEAB398-16| Gerris lacustris
BCEAB399-16| Gerris lacustris
BCEAB420-16| Gerris lacustris
BCEAB432-16| Gerris lacustris
BCEAB178-16| Gerris lacustris
BCEAB183-16| Gerris lacustris
BCEAB184-16| Gerris lacustris
BCEAB186-16| Gerris lacustris
BCEAB386-16| Gerris lacustris
BCEAB185-16| Gerris lacustris
BCEAB395-16| Gerris lacustris
BCEAB391-16| Gerris lacustris
BCEAB392-16| Gerris lacustris
BCEAB387-16| Gerris lacustris

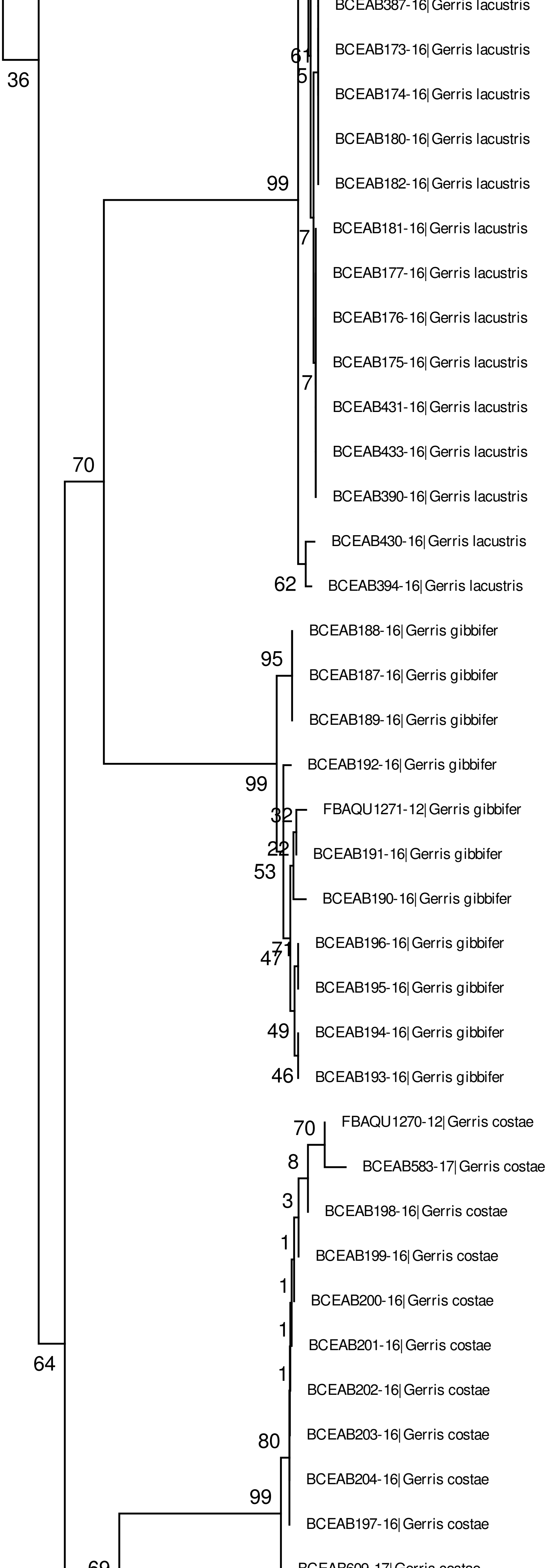
BCEAB387-16|Gerris lacustris
BCEAB173-16|Gerris lacustris
BCEAB174-16|Gerris lacustris
BCEAB180-16|Gerris lacustris
BCEAB182-16|Gerris lacustris
BCEAB181-16|Gerris lacustris
BCEAB177-16|Gerris lacustris
BCEAB176-16|Gerris lacustris
BCEAB175-16|Gerris lacustris
BCEAB431-16|Gerris lacustris
BCEAB433-16|Gerris lacustris
BCEAB390-16|Gerris lacustris
BCEAB430-16|Gerris lacustris
BCEAB394-16|Gerris lacustris
BCEAB188-16|Gerris gibbifer
BCEAB187-16|Gerris gibbifer
BCEAB189-16|Gerris gibbifer
BCEAB192-16|Gerris gibbifer
FBAQU1271-12|Gerris gibbifer
BCEAB191-16|Gerris gibbifer
BCEAB190-16|Gerris gibbifer
BCEAB196-16|Gerris gibbifer
BCEAB195-16|Gerris gibbifer
BCEAB194-16|Gerris gibbifer
BCEAB193-16|Gerris gibbifer
FBAQU1270-12|Gerris costae
BCEAB583-17|Gerris costae
BCEAB198-16|Gerris costae
BCEAB199-16|Gerris costae
BCEAB200-16|Gerris costae
BCEAB201-16|Gerris costae
BCEAB202-16|Gerris costae
BCEAB203-16|Gerris costae
BCEAB204-16|Gerris costae
BCEAB197-16|Gerris costae
36
61
5
99
7
7
70
62
95
99
32
22
53
71
47
49
46
70
8
3
1
1
1
1
80
99
64

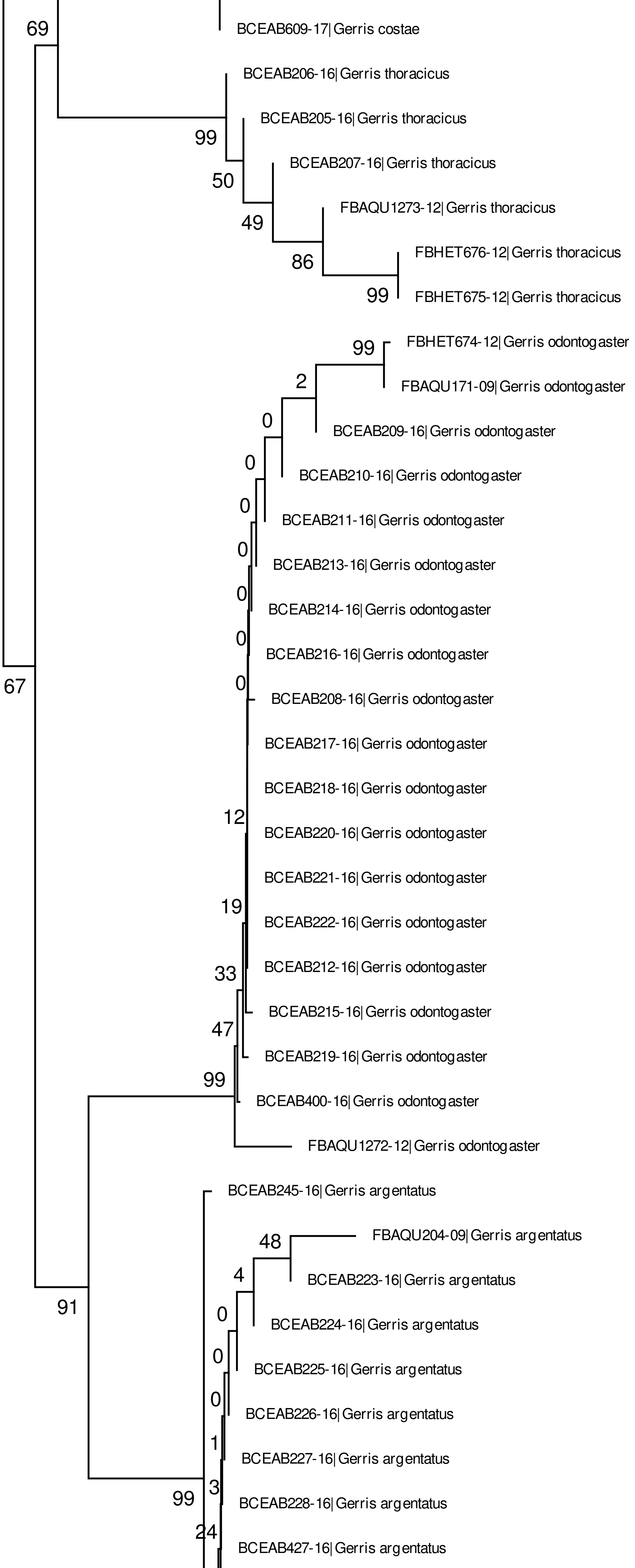

BCEAB609-17|Gerris costae
69
BCEAB206-16|Gerris thoracicus
BCEAB205-16|Gerris thoracicus
99
BCEAB207-16|Gerris thoracicus
50
FBAQU1273-12|Gerris thoracicus
49
FBHET676-12|Gerris thoracicus
86
FBHET675-12|Gerris thoracicus
99
99
FBHET674-12|Gerris odontogaster
2
FBAQU171-09|Gerris odontogaster
0
BCEAB209-16|Gerris odontogaster
0
BCEAB210-16|Gerris odontogaster
0
BCEAB211-16|Gerris odontogaster
0
BCEAB213-16|Gerris odontogaster
0
BCEAB214-16|Gerris odontogaster
0
BCEAB216-16|Gerris odontogaster
67
0
BCEAB208-16|Gerris odontogaster
BCEAB217-16|Gerris odontogaster
BCEAB218-16|Gerris odontogaster
12
BCEAB220-16|Gerris odontogaster
BCEAB221-16|Gerris odontogaster
19
BCEAB222-16|Gerris odontogaster
33
BCEAB212-16|Gerris odontogaster
BCEAB215-16|Gerris odontogaster
47
BCEAB219-16|Gerris odontogaster
99
BCEAB400-16|Gerris odontogaster
FBAQU1272-12|Gerris odontogaster
BCEAB245-16|Gerris argentatus
48
FBAQU204-09|Gerris argentatus
4
BCEAB223-16|Gerris argentatus
91
0
BCEAB224-16|Gerris argentatus
0
BCEAB225-16|Gerris argentatus
0
BCEAB226-16|Gerris argentatus
1
BCEAB227-16|Gerris argentatus
3
99
BCEAB228-16|Gerris argentatus
24
BCEAB427-16|Gerris argentatus

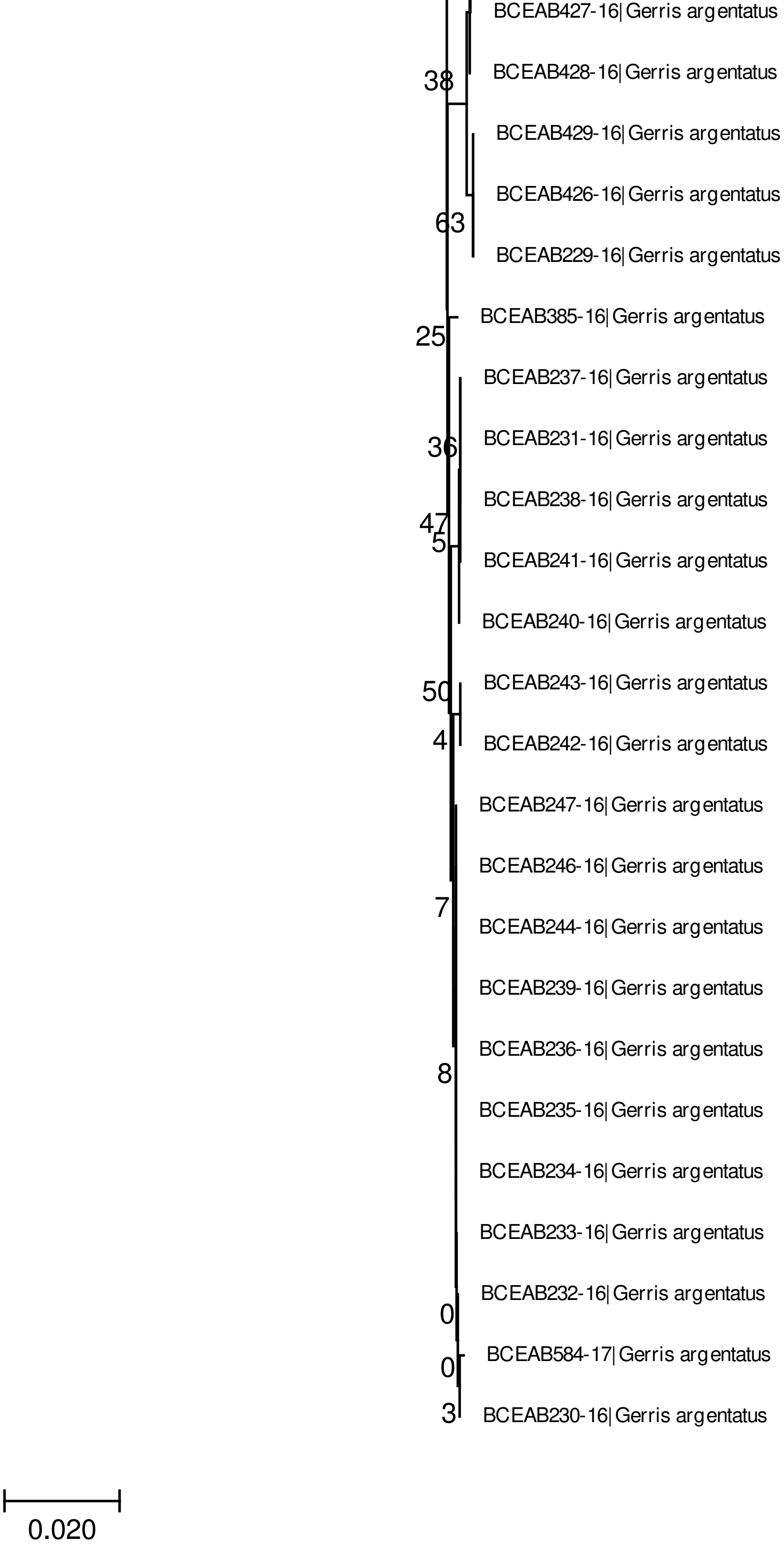

BCEAB427-16|Gerris argentatus
BCEAB428-16|Gerris argentatus
BCEAB429-16|Gerris argentatus
BCEAB426-16|Gerris argentatus
BCEAB229-16|Gerris argentatus
BCEAB385-16|Gerris argentatus
BCEAB237-16|Gerris argentatus
BCEAB231-16|Gerris argentatus
BCEAB238-16|Gerris argentatus
BCEAB241-16|Gerris argentatus
BCEAB240-16|Gerris argentatus
BCEAB243-16|Gerris argentatus
BCEAB242-16|Gerris argentatus
BCEAB247-16|Gerris argentatus
BCEAB246-16|Gerris argentatus
BCEAB244-16|Gerris argentatus
BCEAB239-16|Gerris argentatus
BCEAB236-16|Gerris argentatus
BCEAB235-16|Gerris argentatus
BCEAB234-16|Gerris argentatus
BCEAB233-16|Gerris argentatus
BCEAB232-16|Gerris argentatus
BCEAB584-17|Gerris argentatus
BCEAB230-16|Gerris argentatus
38
63
25
36
47
5
50
4
7
8
0
0
3
0.020
