# Supplementary material for: From water striders to water bugs: the molecular diversity of aquatic Heteroptera (Gerromorpha, Nepomorpha) of Germany based on DNA barcodes
Source: PeerJ. 2018 May 2;6:e4577. doi: 10.7717/peerj.4577 (PMC5936072; doi:10.7717/peerj.4577)
Supplement: Supplemental Information 4 [file peerj-06-4577-s004.docx]

**Supplemental file: Additional References**

Douglas JW, Scott J. 1868. British Hemiptera: Additions and corrections. *Entomologist`s Monthly Magazine* **4**:265–271.

Douglas JW, Scott J. 1869. British Hemiptera: Additions and corrections. *Entomologist`s Monthly Magazine* **5**:259–268, 293–297.

Fabricius JC. 1777. *Genera Insectorum*. Chilonii: Bartsch. [in Latin]

Fabricius JC. 1794. *Entomologia systematica emendate et aucta, secudum classes, ordines, genera, species adjectis synonymis, locis, observationibus, descriptionibus*. Hafniae: Proft. [in Latin]

Fieber FX. 1848. Synopsis aller bisher in Europa entdeckten Arten der Gattung *Corisa*. *Bulletin de la Société Impériale des Naturalistes de Moscou* **21**:505–593. [in German]

Fieber FX. 1860. *Die europäischen Hemiptera. Halbflügler. (Rhynchota Heteroptera)*. Wien: Gerold`s Sohn. [in German]

Fieber FX. 1864. Neuere Entdeckungen in europäischen Hemipteren. B. Neue Arten. *Wiener Entomologische Monatsschrift* **8**:65–86,205–234,321–335.

Heather JM, Chain B. 2016. The sequence of sequencers: The history of sequencing DNA. *Genomics* **107(1)**:1–8.

Herrich-Schaeffer GAW. 1840-1853. *Die Wanzenartigen Insecten – Getreu nach der Natur abgebildet und beschrieben*. Nürnberg: Zehn`sche Buchhandlung. [in German]

Jiu M, Hu J, Wang L-J, Dong J-F, Song Y-Q, Sun H-Z. 2017. Cryptic species identification and composition of *Bemisia tabaci* (Hemiptera: Aleyrodidae) complex in Henan province, China. *Journal of Insect Science* **17(3)**:78. DOI 10.1093/jisesa/iex048.

Leach WE. 1817-1818. On the classification of the natural tribe of insects Notonectides with descriptions of British species. *Transactions of the Linnean Society of London* **12**:10–18.

Linnaeus C. 1758. *Systema naturae per regna tria naturae, secundum classes, ordines, genera, species, cum characteribus, differentiis, synonymis, locis*. Holmiae: Salvii. [in Latin]

Reuter OM. 1880. Nya bidrag till Åbo och Ålands skärgårds Hemipter-fauna. *Meddelanden af Societas pro Fauna et Flora Fennica* **5**:160–236. [in Swedish]

Sahlberg CR. 1819. Dissertatio academica, observationes quasdam historiam notonectidum imprimis Fennicarum, illustrantes propositura. *Aboae*:15. [in Latin]

Yu DW, Ji Y, Emerson BC, Wang X, Ye C, Yang C, Ding Z. 2012. Biodiversity soup: metabarcoding of arthropods for rapid biodiversity assessment and biomonitoring. *Methods in Ecology and Evolution* **3**:613–623.

**Currently missing references that can be added**

Note: All references are first authors of species or genera, so no link to the reference is needed!

Delcourt A. 1909. Recherches sur al variabilité du genre “*Notonecta*”. *Bulletin de la Société Entomologique de Belgique* **43**:373–461.

Fabricius JC. 1775. *Systema entomologiae, sistens insectorum classes, ordines, genera, species, adjectis synonymis, locis, descriptionibus, observationibus*. Flesnburgi & Lipsiae: Kortii. [in Latin]

Fabricius JC. 1790. Nova insectorum genera. *Naturhistorie Selskabet* **1**:213–228. [in Latin]

Flor G. 1860. *Die Rhynchoten Livlands in systematischer Folge beschrieben. Teil 1*. Dorpat: Schulz. [in German]

Horváth G. 1895. Hémipteres nouveaux d`Europe et des pays limitrophes. *Revue de Entomologie* **14**:152–165. [in French]

Hungerford HB. 1928. *Notonecta reuteri*, new name for *Notonecta scutellaris* Reuter 1886. *Bulletin of the Brooklyn Entomological Society* **23**:128.

Müller OF. 1776. *Zoologiae Danicae prodromus seu animalium Daniae et Norvegiae indigenarum. Characteres, nomina, et synonyma imprimis popularium*. Havniae: Hallageriis. [in Latin]

Mulsant E, Rey C. 1852. Description de quelques Hémiptères Hétéroptères nouveaux ou peu connus. *Annales de la Société Linnéene de Lyon* **1852**:76–141,311. [in French]

Schummel TE. 1832*. Versuch einer genauen Beschreibung der in Schlesien einheimischen Arten der Familie der Ruderwanzen Ploteres. Latr*. Breslau: Pelz. [in German]

Stål C. 1861. Nova methodus familias quasdam Hemipterorum disponendi. *Öfversigt af Kungliga Vetenskapsakademiens Förhandligar* **18(4)**:195–212. [in Latin]

Tamanini L. 1947. Contributo ad una revisione del genere *Velia* Latr. e descrizione di alcune specie nuove (Hemiptera Heteroptera: Veliidae). *Memorie della Società Entomologica Italiana* **26**:17–74. [in Italian]

Thomson CG. 1869. Öfversigt af Sveriges Coriser. *Thomson`s Opuscula Entomologica* **1**:26–40. [in Swedish]

Thomson CG. 1871. Bidrag till Sveriges insect-fauna (2). *Thomson`s Opuscula Entomologica* **4**: 361–397. [in Swedish]

Thunberg CP. 1787. *Museum naturalium Academiae Upsaliensis. Part V*. Upsalia: Edman. [in Latin]

Westwood JO. 1833. On the connecting links between the Geocorisae and Hydrocorisae of Latreille, or the land and water bugs. *Magazine of Natural History* **6**:228–229.

Westwood JO. 1834. Mémoire sur les generes *Xylocoris*, *Hylophila*, *Microphysa*, *Leptopus*, *Velia*, *Microvelia* et *Hebrus*; avec quelques observations sur les Amphibiocorisiae de M. Dufour et sur l` état imparfait, mais identique des certaines espèces. *Annales de la* *Société Entomologique de France* **3**:637–653. [in French]
